# Psychiatric comorbidities and sexual health risks in HIV-serodiscordant heterosexual couples involving women with borderline personality disorder: a mixed-method study and theoretical modeling

**DOI:** 10.3389/fpsyt.2026.1775933

**Published:** 2026-04-23

**Authors:** Carlo Lazzari, Jon Rees, Yitka Graham, Rebecca Owens

**Affiliations:** 1 Department of Psychiatry, Intenational Centre for Healthcare and Medical Education (ICHME), London, United Kingdom; 2 School of Psychology, University of Sunderland, Sunderland, United Kingdom; 3 Helen McArdle Nursing and Care Research Institute, Faculty of Health Sciences and Wellbeing, University of Sunderland, Sunderland, United Kingdom; 4 Faculty of Psychology, University of Anahuac, Mexico City, Mexico; 5 University of Huddersfield, Huddersfield, United Kingdom

**Keywords:** borderline personality disorder, complex post-traumatic stress disorder, dependent personality disorder, health behavior, HIV, HIV serodiscordant couples, psychiatry, self-defeating personality disorder

## Abstract

**Background:**

HIV-serodiscordant heterosexual couples consist of one partner who is HIV-positive and the other who is HIV-negative. Our previous studies found that the HIV-negative female partner in no-prevention couples (NPC) may be affected by borderline personality disorder (BPD) and might have a history of child abuse, trauma, and neglect. These couples provide valuable insights into public health, particularly regarding health behaviors and psychosocial factors that influence the relationship between psychopathology, illness behaviors related to borderline personality disorder, and the transmission of sexually transmitted diseases.

**Population and methods:**

This study was conducted in three sequential phases. Phase One involved a cross-sectional, multicenter, anonymous survey of 175 HIV-serodiscordant couples, aimed at assessing preventive sexual behaviors. Participants reported sexual protection use during at-risk sexual encounters on a scale ranging from 0% (‘never’) to 100% (‘always’). Phase Two, which constitutes the core of the present investigation, employed qualitative, unstructured interviews and narrative analysis with HIV-negative female partners in serodiscordant relationships. The analytic focus was on identifying patterns of psychopathological comorbidity and health-related behaviors. Through iterative narrative synthesis and thematic coding, the presence of Borderline Personality Disorder (BPD) emerged consistently across cases. The confirmation of BPD as a clinical diagnosis was thus an outcome of this phase, derived from converging narrative indicators and psychopathological profiles rather than a pre-established inclusion criterion. Phase Three, drawing on the findings from both preceding phases, involved developing a theoretical model of the observed behavioral patterns. Integrating quantitative trends from Phase One with the qualitative insights from Phase Two, we developed a conceptual framework to explain the interaction between BPD-related psychopathology, relational dynamics, and HIV (or other sexually transmitted diseases) risk behaviors. This model aims to guide future healthcare strategies for HIV-negative women with BPD in serodiscordant relationships, a group identified as particularly vulnerable to sexually transmitted HIV due to compromised primary prevention.

**Results:**

Specific health belief models and behaviors related to health emerged among HIV-negative female partners with BPD who decline prevention during behaviors that put them at risk for HIV sexual transmission in stable relationships with HIV-positive males. Results illustrate that women with BPD who have a history of child abuse and trauma often exhibit comorbid self-defeating personality disorder (SDPD), dependent personality disorder (DPD), and complex post-traumatic stress disorder (CPTSD). We could not replicate similar findings in HIV negative males.

**Conclusions:**

The current study confirms that health behaviors and women’s health may be influenced by underlying personality, behavioral, and psychosocial factors, which public health policymakers must address to improve primary prevention of transmissible diseases. We suggest that child abuse, neglect, and trauma may be connected to overlooked health behaviors across a person’s entire life span.

## Introduction

While individual health can be seen as a combination of health literacy and health beliefs (1), recently, there has been increased interest in social and psychopathological factors influencing health behaviors and their decline during pandemics of contagious diseases (2). Social determinants of health are nonmedical factors that include individual traits such as education, income, and health beliefs, as well as social and physical environments such as families, schools, workplaces, neighborhoods, social and biological factors, and the political-economic structure of society (3). Health behaviors are how people influence their own health, either positively or negatively. These actions can be intentional or accidental and may either improve or harm the well-being of the individual or others. In health promotion and prevention, people are active decision-makers (4). According to the Health Belief Model, people are more likely to engage in health behavior if they believe they are susceptible to an illness, perceive it as potentially harmful, and believe there are benefits to the health behavior (5).

However, when the AIDS pandemic and, more recently, the COVID-19 pandemic began, in our infectious diseases and psychiatric clinics, we noticed that health literacy was not enough to prevent people from engaging in harmful health behaviors. The ability to read, understand, and act on health information is referred to as functional health literacy (6). Inadequate health literacy can lead to various negative outcomes, including worsening health, misunderstandings of medical conditions and treatments, difficulties in recognizing and utilizing preventive services, lower self-rated health, reduced adherence to medical advice, increased hospital visits, and higher healthcare costs (7). The current research examines these theories in HIV-serodiscordant couples, defined by the WHO as two people in a stable relationship where one partner is HIV-positive and the other is HIV-negative (7).

In our previous survey of HIV-serodiscordant heterosexual couples (HSDHC) in infectious disease departments and psychiatric settings completed during study phase 1, we discovered that some psychopathological and psychosocial factors influence the decision in couples to decline HIV prevention (NPC) of sexual transmission (8). We called this behavior Samos Syndrome [Sindrome di Samo (It.), El Síndrome de Samo (Sp.), Le Syndrôme de Samo (Fr.) (8)]. In the HSDHC, the female partner who is HIV-negative and has Samos Syndrome might make a mutual decision with her HIV-positive male partner not to use primary prevention to avoid HIV transmission (9). We identified the Samos Syndrome roughly 20 years ago (10). Women with BPD and Samos Syndrome, specifically DP in HSDHC, might choose not to use infection prevention methods during occasional or regular sexual relationships that pose a risk for HIV, despite having sufficient health literacy about the infection (11). We subsequently confirmed that this condition is more common in women with borderline personality disorder and a history of childhood trauma, sexual abuse, and attachment issues (12). Samos Syndrome refers to the tendency of some persons—often with a history of childhood violence, abuse, neglect, or trauma—to form romantic, emotionally intimate, and stable sexual relationships with partners who present with significant and debilitating physical, mental, or social conditions (13). The behavior in question was called “Samos Syndrome” by the authors, based on the story of Zambaco Pacha, a Turkish doctor who visited many leprosaria worldwide in 1800, including one on the Greek island of Samos (13). According to Zambaco Pacha, Samos Island in Greece was a place where people with leprosy could participate in community activities. They were also allowed to marry members of the local community (14). So, it turned out that a woman developed feelings for a man who was highly contagious and suffering from leprosy and bubonic plague (15). This episode is reported in Guido Ceronetti’s book, *The Silence of the Body* (15). Hence, in Samos Syndrome emotional dysregulation and impulsivity may heighten vulnerability to risky health behaviors (8). Samos Syndrome could be considered as a psychological mechanism that contributes to omitted prevention during pandemics, particularly among individuals who either minimize danger or paradoxically neglect prevention of infectious diseases, in this case, HIV (16, 17).

The health model adopted in the current study is the biopsychosocial model that conceptualizes health and illness as emergent outcomes of interacting biological mechanisms, psychological processes, and social contexts (18, 19). At the individual level, evidence suggests that the intersection of trauma and personality dysfunction creates a “perfect storm” for infectious disease transmission modulated by an “attachment-risk” trade-off in women with BPD (20). Our former research found that when biological stress systems (e.g., pandemics or risk of infections during lockdowns) are over-activated by trauma history, the social need for proximity often renders biological survival secondary (2). This physiological vulnerability aligns with findings that individuals with higher relational instability often experience “hyper-activation” of attachment needs during health crises, leading to a neglect of protective health behaviors (21). A critical psychological component often overlooked in traditional health education is the role of mentalization, that is, the ability to understand the mental states of oneself and others (22). Research indicates that under the stress of romantic or caregiving roles, women with BPD may lose the capacity to appraise partner-related dangers, a phenomenon known as “collapse of relational foresight” (23). This collapse can explain why personality can be a predictor of unprotected sexual behavior among people living with HIV/AIDS (24).

The social dimension of our model is further supported by the “syndemic” theory of health, which posits that social instability, trauma, and disease do not exist in isolation but cluster and interact (25). In serodiscordant pairings, the social pressure to prove “undying loyalty” can lead to what has been termed “negotiated safety,” which often breaks down into unprotected intimacy when relational boundaries erode (26). This behavior provides a social context for why terminal health risk exposure remains high despite clinical interventions that focus only on individual behavior rather than the relational system (**Table 1**).

**Table 1:** Applied biopsychosocial model of health risk exposure in BPD populations.

Domain	Biopsychosocial component	Research application and findings
Biological	Physiological vulnerability	Identifies the terminal link between relational collapse and physical exposure to pathogens (e.g., HIV/COVID-19).
Psychological	Internalized trauma & affect	Explores how trauma history drives “low mood” and emotional dysregulation, which impairs cognitive appraisal of risk.
Social	Relational dynamics	Identifies specific “Romantic” and “Caregiving” pathways where boundary erosion and role confusion lead to relational instability.
Integration	The “Cascade” effect	Demonstrates how these domains converge, showing that psychological “loyalty” and symbolic intimacy override biological “self-preservation.”

Regarding the HIV infection, it progressively weakens the immune system by targeting CD4 T cells and may lead to AIDS if untreated, with AIDS marked by severe immune deficiency and increased susceptibility to infections and cancers; being HIV-positive means the virus is detectable through antibodies, antigens, or viral RNA, though not necessarily symptomatic or indicative of AIDS; furthermore, transmission occurs via specific infector’s body fluids, blood, semen, vaginal fluids, rectal secretions, and breast milk, from individuals with a detectable viral load, entering the recipient bloodstream through mucous membranes, open wounds, or injection (27–32).

In DSM-5, the criteria for diagnosing BPD include (1) desperate attempts to avoid perceived or actual separation; (2) a passionate and unpredictable pattern of relationships that switch between extremes of idealization and devaluation; (3) identity disturbance: a notable and ongoing unstable perception of self-awareness and self; (4) at least two potentially harmful impulsive behaviors, such as overspending, drug misuse, reckless driving, sexual activity, binge eating, and others; (5) significant emotional hypersensitivity, such as intense episodic dysphoria, anxiety, or irritability, lasting a few hours or rarely more than a few days, leading to affective instability; (6) persistent feelings of emptiness; (7) inappropriate, intense, or hard-to-control anger, such as frequent outbursts, persistent rage, or recurring violent conflicts; and (8) brief paranoid thoughts or acute dissociation (33).

## Research aims

This study examines the intersection of borderline personality disorder (BPD), psychiatric comorbidities, and health risk behaviors in women involved in challenging romantic relationships. It focuses on how relational dynamics may increase vulnerability to HIV and Sexually Transmitted Diseases (STDs), particularly through intentional self-harm and neglect of preventive health measures. Key comorbidities, such as self-defeating personality disorder, dependent personality disorder, and complex PTSD, were empirically explored within this subgroup. Although BPD was not an initial selection criterion, it progressively emerged as the predominant diagnosis, confirmed via ICD-10/11 and DSM-5/DSM-5-TR based clinical interviews. This diagnostic outcome subsequently shaped the thematic analysis and validated the study’s focus on BPD and its associated behavioral risks. We clarify that while aspects of Samos syndrome have been reported in previous publications, the results presented in the current manuscript are entirely novel. This study represents a condensation and theoretical development of our earlier work, as cited in the references, but the methods, conclusions, and integrative framework are presented here for the first time. The manuscript builds upon prior findings to offer a unified analysis and original interpretation that has not been published elsewhere. We have revised the relevant statement to make this distinction clearer.

## Methodology and methods

### Diagnostic frameworks

Childhood experiences of emotional neglect, physical or sexual abuse, and invalidating caregiving environments are consistently identified as key developmental precursors to BPD. These relational traumas often disrupt attachment formation and emotional development, leading to enduring difficulties in affect regulation and interpersonal functioning. Our biosocial model of BPD suggests that individuals with heightened emotional sensitivity, when raised in chronically invalidating contexts, are particularly vulnerable to developing the disorder. Empirical research supports this framework, showing strong associations between early maltreatment and the emergence of core BPD features, including unstable relationships, identity disturbance, and impulsivity (34–36). Behavioral traits indicative of BPD are defined according to DSM-5 and DSM-5-TR criteria, which describe a pervasive pattern of instability in relationships, self-image, affect, and impulsivity. A diagnosis requires five or more of nine symptoms, including abandonment fears, unstable relationships, identity disturbance, impulsivity, self-harm, affective instability, chronic emptiness, intense anger, and transient paranoia or dissociation (37, 38). Participants were assessed using ICD-10 and ICD-11 diagnostic categories (World Health Organization, 1992; 2019), which reflect the standards used in our clinical setting and facilitate international comparison (39, 40). While the ICD framework guided formal diagnostic classification, we also used DSM-5 criteria for Borderline Personality Disorder (American Psychiatric Association, 2013) to shape the conceptual framework of the study (41). This dual-system approach was necessary because certain features central to BPD, such as affective instability, identity disturbance, and interpersonal dysfunction, are more clearly described in DSM-5 than in ICD-10/11.

Another psychopathology reported in the current study is *Trauma-Reenactment Syndrome* (TRS), which reflects an unconscious drive to recreate elements of earlier traumatic experiences, often through relationships, behaviors, or life choices that mirror the original harm. This pattern is understood as an attempt to gain mastery over overwhelming past events, yet it frequently results in revictimization, repetition of problematic dynamics, and chronic emotional distress. The phenomenon is closely linked to dysregulated attachment, dissociation, and the way traumatic memory is stored somatically and procedurally rather than narratively. Contemporary trauma theory emphasizes that reenactment is not a sign of choice or weakness but a neurobiological and psychological survival adaptation that becomes maladaptive when repeated outside the original context (42, 43).

*Self-Defeating Personality Disorder* (SDPD) can be understood as a coherent pattern in which a person repeatedly engages in behaviors that undermine his or her own well-being, gravitating toward disappointment, criticism, or instability even when healthier alternatives are available. These behaviors are shaped by early relational experiences that create enduring negative self-schemas, fostering a deep sense of not deserving success, care, or stability. As these beliefs take hold, they contribute to chronic self-sabotage, where individuals disrupt progress or relationships in ways that recreate familiar disappointment, even when doing so conflicts with their stated goals. Alongside this, forms of attachment insecurity develop, leading people to seek relationships that confirm negative expectations or to reject supportive ones because safety and stability feel unfamiliar or emotionally threatening. Over time, these dynamics become trauma-linked patterns, in which behavioral repetition reflects earlier relational or developmental adversity rather than conscious choice. Although the diagnostic label was removed from later editions of the DSM, the underlying patterns remain clinically relevant, especially in trauma-informed psychotherapy, where they are understood as learned survival strategies that once protected the individual but later became restrictive and harmful (44, 45).

*Complex PTSD* (CPTSD) can be understood as a pattern of long-lasting psychological and relational difficulties that emerge after prolonged, repeated, or inescapable trauma, where the effects extend beyond those seen in PTSD and become woven into a person’s sense of self, emotions, and relationships. People often struggle with emotional dysregulation, finding it difficult to manage overwhelming anger, shame, fear, or emotional numbness, and these reactions can feel sudden, intense, or out of proportion to current circumstances. Alongside this, a deeply entrenched negative self-concept may develop, marked by persistent feelings of worthlessness, failure, or being permanently damaged, often rooted in the way chronic trauma shapes identity and self-beliefs. Interpersonal life is also affected: chronic interpersonal disturbances such as mistrust, fear of closeness, or repeated involvement in unsafe or unstable relationships reflect how trauma disrupts attachment and expectations of others. These difficulties become trauma-linked patterns, where emotional and behavioral responses are shaped by earlier experiences of entrapment or threat rather than by present-day realities (46, 47).

*Dependent Personality Disorder* (DPD) is defined in the DSM-5-TR as a pervasive and excessive need to be taken care of, leading to submissive, clinging behavior and fears of separation, beginning by early adulthood and present across many contexts. Diagnosis requires five or more characteristic features, including difficulty making everyday decisions without reassurance, needing others to assume responsibility for major life areas, fear-based avoidance of disagreement, difficulty initiating tasks due to low self-confidence, going to excessive lengths to obtain nurturance, feeling helpless when alone, urgently seeking new relationships when one ends, and being preoccupied with fears of being left to care for oneself. DPD sits within Cluster C (anxious/fearful) personality disorders, and its presentation is shaped by cognitive beliefs of helplessness, behavioral patterns of dependency, and emotional vulnerability to abandonment (48, 49).

### Research design

We used a convergent parallel mixed-methods design integrating quantitative prevalence data with qualitative insights to examine psychiatric comorbidities, risk behaviors, and health vulnerabilities in women with borderline personality disorder (BPD) in high-risk relational contexts. This simultaneous analysis enhances validity through triangulation and supports both empirical and interpretive outcomes, thereby increasing clinical and public health relevance (50). Structured instruments, open-ended survey items, and ethnographic observation were used to generate both measurable trends and contextual insights. NVIVO software (51) supported narrative analysis, while quantitative data were managed in Excel and elaborated using MedCalc (52). This approach enabled triangulation and enriched interpretation, particularly suited to healthcare and education contexts, and is essential for understanding both outcomes and underlying processes (53–59).

The study employed a mixed-methods design to triangulate data and generate a layered, reflexive understanding of complex phenomena in mental health practice. Each qualitative method contributes a distinct lens: (1) *narrative analysis* explored the storied nature of experience, privileging temporality, identity, and meaning-making; (2) *thematic analysis* identified patterns across data using a theory-driven approach informed by DSM-5, ICD-10/11, and psychodynamic models, allowing for structured coding while remaining open to emergent insights; (3) *grounded theory* supported theory generation from data, revealing latent processes and mechanisms; and (4) *ethnography* situates individual narratives within broader cultural and organizational contexts, particularly when direct interviews were not feasible (60–64). This hybrid strategy aligns with what Bella Williams describes as *hybrid qualitative analysis*, where thematic analysis is enriched by integrating narrative and ethnographic insights to produce a more nuanced and layered interpretation (65). Similarly, Creswell suggests that combining qualitative methods can enhance the depth and breadth of inquiry, especially when addressing multifaceted social phenomena (66). Integrating qualitative approaches into a mixed-method framework enhances validity through triangulation, provides distinct insights via complementarity, informs coding structures through developmental sequencing (e.g., narrative analysis informing thematic analysis), and situates findings in real-world contexts via contextualization (e.g., ethnographic data). This aligns with integrative evidence synthesis models that layer qualitative methods to refine theoretical constructs and interventions (67).

### Interviews protocols

#### Data collection and surveys

This study employed a mixed-methods approach combining structured surveys, psychiatric interviews, and open-ended questions to explore sexual protection use, relationship history, education, and parental attachments among HIV-serodiscordant couples. NVIVO software supported thematic analysis of transcripts, identifying patterns within psychopathological frameworks for BPD. Structured interviews ensured consistency and comparability, while open-ended and unstructured interviews captured nuanced, experiential data. This dual strategy enabled both statistical mapping and interpretive depth, particularly in understanding how early relational trauma and psychoeducational histories influence risk-taking behaviors. The integration of ethnographic observation further enriched contextual insights, aligning with best practices in healthcare and education research (56–58, 61, 68–73). In Phase 1, structured surveys used standardized, closed-ended questionnaires to assess HIV-related knowledge, attitudes, and behaviors among target HIV-serodiscordant couples, enabling consistent data collection and quantitative analysis. These were followed by structured psychiatric interviews employing validated tools to both explain non-adoption of HIV prevention and generate psychiatric diagnoses, serving diagnostic and explanatory roles within the mixed-methods design. In later phases, unstructured interviews provided open-ended, participant-led narratives that captured lived experiences and emergent themes around HIV prevention, relationships, and mental health, offering contextual depth beyond structured formats.

#### Study phases, participants, and settings

##### Phase 1: (1994–2009)

This phase adopted a cross-sectional quantitative survey with HIV-sero-discordant couples across multiple centers. Phase 1 aimed to investigate the objectives underlying biopsychosocial dynamics in HSDHC, with a focus on individuals diagnosed with BPD. The data collection combined structured surveys and informal interviews to capture both statistical trends and experiential narratives. A total of 175 couples were recruited using purposive sampling across international sites. Interviews explored clinical histories, relational stressors, and mental health profiles. The settings included HIV clinics, genitourinary medicine units, and community psychiatric teams, ensuring ecological validity and a comprehensive lens on the interplay between psychiatric comorbidity and HIV transmission risk.

##### Phase 2: (2009–2019)

This phase involved a qualitative, exploratory study with unstructured interviews and narrative analysis, focusing on the phenomenology of Samos Syndrome. Phase 2 (2009–2019) was a qualitative, exploratory study investigating the objectives of relational and psychological experiences in HSDHC, with a focus on the phenomenology of Samos Syndrome—characterized by relational ambivalence, identity fragmentation, and emotional dysregulation. Data collection involved unstructured interviews and narrative analysis, guided by Braun and Clarke’s Thematic Analysis across seven iterative stages. Transcripts were anonymized and analyzed inductively to identify emergent themes reflecting emotional, interpersonal, and diagnostic complexities. The settings mirrored Phase 1, spanning HIV clinics, genitourinary medicine units, and community psychiatric teams, ensuring diverse relational contexts and integrated psychiatric-sexual health perspectives. The participants were the same individuals who were in follow-up arms in HIV clinics; one member of the 175 HSDHC was identified as Samos, and thus a total of 80 participants.

##### Phase 3: (2019-2025)

The design was a theory development and synthesis from previous phases, including transcript reviews and final interpretations. Phase 3 focused on objectives of theory development and synthesis, advancing understanding of relational psychopathology and health vulnerability in HSDHC, particularly those with borderline personality disorder (BPD) and Samos Syndrome. The data collection drew exclusively from previously gathered transcripts, thematic matrices, and analytic memos from Phases 1 and 2. Using abductive reasoning and set-theoretic modeling, we mapped comorbidities across BPD, self-defeating personality disorder (SDPD), dependent personality disorder (DPD), and complex post-traumatic stress disorder (CPTSD). The settings involved academic and analytic environments, with interdisciplinary collaboration across psychiatric and qualitative research teams, maintaining continuity with the multicenter framework established in earlier phases. Set theory and path diagrams provided a formalized and visually intuitive framework for modeling theoretical constructs in research, enabling the representation of intersecting psychological, behavioral, and relational domains with conceptual clarity and structural coherence. Applications of Set Theory in Phase 3 were as follows:

- Conceptual mapping Sets allow researchers to define and compare categories such as diagnostic groups, behavioral traits, or relational dynamics. For example, BPD, DPD, and trauma histories can be treated as sets whose intersections reveal clinically significant patterns. *Narrative Structuring*, where sets are used to organize narrative data into typologies, where each set represents a thematic or diagnostic domain. This supports both idiographic depth and thematic abstraction (74).
- omorbidity and overlap *using formal logic integration* (e.g., *BPD*∩*DPD*∩*SDPD*∩*TRS*∩CPTSD), researchers can formally represent how multiple conditions or experiences co-occur, offering clarity in understanding complex syndromes or behavioral profiles using logic expressions from set theory (75).
- Path Diagrams: These visual tools, derived from set theory, help illustrate shared and exclusive attributes across constructs. They are especially useful for communicating theoretical models, such as relational addiction emerging from overlapping vulnerabilities. These paths were extracted from beta β coefficients.

#### Rigor of the study

The study followed the criteria established by Guba and Lincoln (76) to ensure transferability, dependability, credibility, and confirmability. The findings were firmly grounded in the collected data and underwent audits across multiple centers to enhance reliability. Theories and conclusions aligned with the current understanding of psychopathology, using precise terminology and concepts. Researcher bias was mitigated through data triangulation, the sharing of findings, and feedback from subject-matter experts, as well as blogs, media, and colleagues. Dependability was confirmed as independent researchers worldwide identified Samos Syndrome in their studies and discussions. Transferability was achieved by replicating the study in different geographic locations, which consistently produced similar phenomena (76).

#### Research positionality

The authors acknowledge their positionality as experienced clinicians and researchers, shaping the lens through which this study was conducted. CL brings dual postgraduate training in psychiatry and infectious diseases, offering integrated expertise in mental health and HIV care. The other authors contributed a strong foundation in population health and service evaluation. Their combined experience in HIV-related research, particularly its psychological and psychiatric dimensions, provides deep insight into both clinical realities and the structural determinants of health. Grounded in a commitment to social justice, equity, and compassionate care, their work seeks to enhance service quality and address systemic barriers to health and behavior.

#### Methodology, theoretical frameworks, and theory construction

The current research adopted a constructivist approach as outlined by Guba and Lincoln (1984), emphasizing the locally constructed nature of reality (60). The epistemological framework was transactional, meaning that knowledge was shaped through interactions between researchers and participants, with patient narratives interpreted through the researchers’ subjective lenses. Methodologically, the study employed a hermeneutic and dialectical approach, focusing on the interpretation and reconstruction of social constructs to develop theories that explain observed phenomena (76, 77).

We adopted an ontological perspective to explore the nature of reality, which is shaped by locally and individually constructed views. The epistemology examined the relationship between the knower (researcher) and the subjects being understood, portraying it as a transactional process. This included collecting patients’ narratives alongside researchers’ subjective interpretations of the phenomenological worlds (78). Since the constructivist ontology is relativistic, we identified multiple realities that vary across social, psychological, and interpersonal factors. Our approach was hermeneutic and dialectical, and social constructions were strengthened through our interactions with the target population. Therefore, our inquiry focused on understanding and reconstructing to develop theories about the observed phenomena (79).

We primarily used the WHO’s ICD-10/11 but also DSM-5/5TR diagnosis categories. This study employs a Grounded Theory approach, conducting participant observations and semi-structured interviews to gain a deeper understanding of the phenomenon of interest. Through narrative analysis, themes and sub-themes were identified, which informed the development of conceptual frameworks. The findings contribute to both middle-range theories, providing explanations specific to particular contexts, and grand theories, which offer broader theoretical insights (80). We used the Borsboom et al. theory construction model, where developing a theory involves five stages (81) (**Table 2**).

**Table 2:** Borsboom et al. (81) theory construction stages and our naturalistic findings (see (66).

Step (64)	*Our phenomenological observations and grounded theory*
1. Identify empirical phenomena	Women with BPD who are HIV-negative engaged in unprotected and consensual sexual relationships with HIV-positive male partners.
2. Formulating a prototheorid through abductive reasoning	HIV sexual transmission and contagion are not always accidental, as shown by our observations in HIV-serodiscordant couples.
3. Formalize theory and phenomena	Women diagnosed with BPD who are HIV-negative and engage in consensual, unprotected sexual relationships with partners who have HIV/AIDS often report experiences of childhood trauma and abuse.
4. Checking explanatory adequacy	Disruptions in early attachment during childhood can lead to changed health behaviors throughout a person’s lifespan.
5. Assessing the overall worth of the theory	By triangulating naturalistic observations with targeted surveys, women with BPD report that early parenting attachment characterized by problematic parents is linked to suboptimal health behaviors from childhood to adulthood, along with inverse relationships to socioeconomic variables. Notably, women with higher education and wealth exhibit fewer protective behaviors in HIV serodiscordant couples.

#### Data analysis

*Phase 1 of the study* included a cross-sectional anonymous survey of HIV-serodiscordant couples. We asked the couples, ‘With 100% being ‘every time you have a sexual relationship,’ how often do you use sexual protection for HIV prevention?’. The study was conducted in infectious disease departments and community psychiatric teams. The first author (CL) performed the interviews, which were later verified through multidisciplinary team discussions to confirm and triangulate the diagnostic hypotheses. The statistical analysis employed rigorous methods to ensure the reliability of the observed differences in sexual protection use across sociodemographic groups. Confidence intervals were estimated using the Wilson score method, which offers improved accuracy for binomial proportions, particularly in samples of moderate size. To assess the significance of differences between paired groups, two-proportion z-tests were conducted. All comparisons yielded statistically significant results at the conventional alpha level of 0.05, with p-values consistently below 0.001. To elaborate on the findings in Phase 1, we employed path analysis to examine direct and indirect associations among study variables within a theoretically specified causal framework. Standardized beta (β) coefficients were estimated using ordinary least squares regression, implemented in *AMOS* (Analysis of Moment Structures). The model assumed linearity, additivity, and absence of measurement error. Model fit was evaluated using conventional indices. This approach enabled quantification of mediating pathways and assessment of hypothesized structural relationships relevant to the study context (82, 83).

In Phase 2, we carried out a narrative analysis of interviews with HIV-serodiscordant couples (see Appendix). Braun and Clarke’s Thematic Analysis involves seven stages. These stages typically include: (1) *familiarization with the data*: diving into the dataset by reading and rereading transcripts while noting initial ideas; (2) *generating initial codes*: finding meaningful segments of data and systematically assigning codes; (3) *searching for themes*: *grouping related codes* into broader themes that reveal patterns in the data; (4) *reviewing themes*: refining themes to ensure they align well with the dataset; (5) *defining and naming themes*: explaining the core of each theme and its relevance to the research question; (6) *producing the report*: writing a structured analysis that connects themes with supporting data; and (7) *reflexivity and interpretation*: critically reflecting on the researcher’s role and how it influences the findings (84). To examine relational trajectories and behavioral outcomes associated with Samos Syndrome, the study used a hybrid analytic strategy combining narrative analysis with path modelling. This integrative approach allowed qualitative depth to be translated into structural relationships, supporting a constructivist and pragmatic epistemology. Narrative constructs, such as educational incongruence, parental relationship quality, and emotional dysregulation, were coded and operationalized into variables within a matrix-based path model. Independent, mediating, and dependent variables were mapped to explore relational plausibility rather than causality. Standardized beta coefficients quantified associations, thereby identifying mechanisms that shape health-risk behaviors and informing clinical and psychosocial interventions (85–89).

In Phase 3, findings from earlier phases informed the development of both middle-range theories, which offer context-specific, empirically grounded explanations, and grand theories, which provide broader conceptual frameworks applicable across disciplines. Middle-range theories clarified mechanisms within defined settings, while grand theories addressed overarching patterns in human behavior and relational dynamics. Using Grounded Theory, the study employed participant observations and semi-structured interviews to inductively generate themes and subthemes. This iterative process enabled the emergence of conceptual frameworks, integrating contextual insights and experiential narratives to build a comprehensive understanding of relational psychopathology and health vulnerability (63, 90–92).

When direct unstructured interviews were impractical, we adopted a naturalistic ethnographic approach, gathering data through informal, work-based interactions. This method aligns with *symbolic interactionism* and *situated learning theory*, emphasizing meaning-making through real-world engagement. Drawing on Lazzari’s ethnographic work in medical settings, we collected narratives from individuals exhibiting signs of Samos Syndrome and reflections from their HIV-positive partners. To analyze these accounts, we applied a dual-framework thematic analysis that combined *Grounded Theory* and *Braun and Clarke’s reflexive approach*, allowing for inductive theme development and interpretive depth. We also examined educational and familial histories to identify patterns of trauma, later confirmed through interdisciplinary team discussions across centers (93–97).

#### Use of vignettes in clinical education

Vignettes are valuable tools in medical education and research, offering realistic yet controlled scenarios that promote ethical reflection, clinical reasoning, and inclusive learning. They support qualitative inquiry and protect patient confidentiality by anonymizing real cases. Used in narrative-driven training, vignettes enhance engagement, critical thinking, and cultural sensitivity. Their structured format allows educators to assess decision-making and diagnostic skills while fostering a deeper understanding of complex clinical situations. Overall, vignettes improve the validity of qualitative data collection and align educational practices with ethical and empirical standards (98–101). Rather than directly merging transcripts with fictional vignettes, we followed a multi-stage interpretive process. Individual narratives from participants with similar clinical profiles were first analyzed separately and then synthesized into composite vignettes that represent recurring emotional, relational, and behavioral patterns. Drawing on narrative synthesis and interpretive phenomenology, these fictionalized constructs preserved the integrity of lived experience while distilling complex data into coherent forms. Early narrative analysis informed their structure and meaning-making, and thematic analysis of the unified typology revealed cross-cutting patterns. This approach supports methodological pluralism, balancing idiographic depth with thematic abstraction and ensuring epistemic transparency throughout the analytic process (102, 103, 104).

#### Ethical consideration

The study adhered to rigorous ethical standards, including the Helsinki Declaration, with all data anonymized and collected through confidential interviews and structured assessments conducted by one of the authors (CL). Participants, particularly HIV-serodiscordant couples, were informed of their right to withdraw and provided consent under a clearly defined ethical framework. The study followed the ethical standards set by the relevant institutional and national committees on human experimentation, as outlined in the Helsinki Declaration (World Medical Association, 2013) (105). All surveys were anonymized and conducted through confidential interviews via AIDS counseling hotlines, ensuring participant confidentiality and protection (106). Additionally, structured interviews were conducted by one of the authors (CL). To protect confidentiality and uphold ethical standards, the narratives in this study were created by synthesizing real cases and typical accounts. Ethical approval was secured from the participating couples and relevant healthcare organizations, and all data were fully anonymized before analysis. The right to withdraw was a condition explicitly communicated to all participants during the consent process to ensure informed participation. All eligible individuals, meeting the study’s inclusion criteria, were required to understand and acknowledge this right as part of the ethical framework underpinning the study.

To protect anonymity, accounts from multiple individuals were merged into fictional vignettes, maintaining the original meaning while ensuring confidentiality. Storytelling, as defined by Hanna Meretoja in The Ethics of Storytelling (2018), is described as a morally complex interpretive practice that goes beyond sharing experiences to actively shape ethical understanding and social possibilities (107). Drawing on narrative hermeneutics, Meretoja identifies six ethical dimensions that support storytelling: fostering the imagination of alternative futures, enabling self-understanding, facilitating nuanced comprehension of others, reflecting transitional spaces between narratives, enhancing perspective-taking, and serving as ethical inquiry (89). Meretoja argues that narratives are not ethically neutral but are influenced by historical and cultural contexts, capable of both reinforcing dominant ideologies and encouraging critical change (89). This ethical framework emphasizes the relational nature of storytelling and the responsibilities associated with narrative acts, particularly in research, education, and public discourse. By developing a theory in which storytelling affects moral imagination and fosters dialogical engagement, Meretoja views narrative as a space for reflective ethical practice and social critique (89). All collected data, including audio recordings and written transcripts, were securely stored and later destroyed after the analysis was completed (108).

Participants, especially members of HIV-serodiscordant couples, responded to open-ended questions designed to complement a structured psychiatric interview. These questions explored sexual protection use in sexual relationships, previous partnerships, educational background, and parental dynamics, topics previously identified in structured surveys as important factors influencing behavioral patterns and psychoeducational backgrounds (see Appendix; see 109).

Transcripts were systematically reviewed to identify key themes and subthemes, following Braun and Clarke’s six-phase approach to thematic analysis (110). The process started with familiarization, during which transcripts were carefully read and re-read to ensure a deep engagement with the data. Initial codes were created to identify meaningful patterns, which were then systematically grouped into broader themes and subthemes (56). Narratives were analyzed within established BPD frameworks, ensuring a structured and rigorous approach to thematic categorization. Thematic clustering was performed only when BPD was thoroughly confirmed as the main factor influencing the observed behaviors and narratives, maintaining validity and coherence in the analysis (111).

#### Conversion of qualitative narratives to quantitative path estimates

This study adopted a convergent mixed-methods analytic design, integrating qualitative narrative synthesis with quantitative effect-size estimation through a structured process of quantifying *qualitative data*. The aim was to translate qualitative relational judgments into interpretable quantitative parameters while preserving theoretical meaning and analytic rigor, consistent with established mixed-methods methodology (3, 42, 112). The initial stage involved a rigorous thematic analysis of the semi-structured interviews. Narrative segments were evaluated against the theoretical constructs of the biopsychosocial cascade. To move from text to path estimation, we applied a standardized coding protocol: (1) Thematic Density: Constructs were assigned values based on the frequency and intensity of their occurrence within the narrative, (2) Ordinal Scaling: Variables were scored on a 3-point scale (0 = absent, 1 = moderate, 2 = strong presence) to capture the severity of traits, and (3) Binary Mapping: specific were coded as binary markers (0 = not present, 1 = present).

The analytic process further unfolded in four sequential stages: (1) narrative synthesis of qualitative material, (2) independent relational strength ratings by four assessors, (3) aggregation of Pearson’s correlation coefficients (*r*) across assessors, and (4) transformation of aggregated *r* values into standardized beta coefficients (β) for multivariate modeling. The following stages were followed. In stage 1, we adopted a narrative synthesis and identification of relational patterns. Qualitative data and narratives were first analyzed using narrative synthesis to identify recurring relational patterns between core constructs (e.g., BPD traits and clinical or behavioral outcomes). Rather than counting isolated codes, relationships were interpreted within their contextual and theoretical meaning. In Stage 2, independent qualitative-to-quantitative ratings were conducted by four professionals with expertise in psychopathology and mixed-methods research, who independently reviewed the synthesized narratives and assigned qualitative relational-strength judgments (e.g., “no,” “low,” “medium,” and “high association”). Each assessor then mapped these judgments onto Pearson’s correlation effect-size ranges using established conventions (4, 6). This step reflects a quantifying procedure in which qualitative relational assessments are systematically translated into numerical indicators while retaining interpretive grounding (101). In Stage 3, we aggregated Pearson’s *r* values across assessors. For each construct pair (e.g., ‘BPD-Mood’), the four independently assigned *r* values were averaged to produce a single consensus Pearson’s correlation coefficient. Averaging across assessors reduced individual bias and increased the stability of the estimated relational strength, functioning analogously to aggregation procedures used in inter-rater consensus models. The resulting mean *r* values were treated as integrative effect-size estimates, suitable for subsequent modeling and interpretation. In Stage 4, the aggregated Pearson’s *r* values were then transformed into standardized beta coefficients (β). In cases where a single predictor was modeled, β was equivalent to *r*. In multivariate models, β coefficients were derived using established partial-correlation formulas, reflecting the unique contribution of each predictor after accounting for shared variance (4) (**Table 3**).

**Table 3:** Practical examples of qualitative assessment, averaged *r*, and β transformation.

Qualitative judgement	Clinical example	Narrative rationale (examples)	Pearson’s *r* (per assessor example)	Mean *r* (4 assessors)	Example standardized β	Transformation formula^a^
No correlation	BPD traits ↔ euthymic mood	Stable euthymia shows minimal linkage with core BPD pathology	.04,.06,.05,.05	.05	β = .05	Simple model: β = r
Low correlation	BPD ↔ drug use	Substance use occurs variably in BPD but is not defining	.15,.18,.20,.17	.18	β ≈.12	Multiple model: $\;\beta \;=\;(r_{\gamma x}\;-\;r_{\gamma }\cdot zr_{x}z)/(1\;-{\text{ r}}_{\text{x}}z²)$
Medium correlation	BPD ↔ obsessive-compulsive disorder	OCD co-occurs in a meaningful but non-central subset	.35,.40,.38,.39	.38	β ≈.27	Partial-correlation-based β
High correlation	BPD ↔ self-harm	Self-harm is a core behavioral feature of BPD	.60,.64,.63,.61	.62	β ≈.51	β ≈ r when predictor dominance is high

^a^ β values are expected to be attenuated relative to r in multivariate models due to shared variance among predictors.

These values formed a correlation matrix that AMOS used to estimate how trauma-related constructs predicted later risk-taking. The transformation from Pearson correlations to standardized beta coefficients followed the rule that, when variables are standardized, the simple regression coefficient equals the correlation, such as β = *r.* For models with multiple predictors, AMOS adjusted each coefficient to reflect its unique contribution. With *y* representing the outcome variable we are trying to predict (e.g., in our study, this could be something like Risk−Taking, Unprotected Intimacy, or Health−Risk Exposure), while *x* = the predictor variable whose influence we want to estimate (e.g., Trauma History, Symbolic Intimacy, Impaired Risk Appraisal, or Relational Instability). In this case, for the multiple-predictor case the formula becomes:


$$\beta_{x}=\;r_{yx}-\sum r_{xk}\beta_{k}$$

Here, *r_yx_* represents the correlation between the predictor *x* and outcome *y, r_xk_* the correlations between the predictor *x* and other predictors, and *β_k_* beta coefficients of those other predictors.

Beta coefficients β were chosen because they place all predictors on a common scale, allowing direct comparison of the relative strength of each psychosocial construct within the cascade while preserving the meaning of participants’ narratives.

## Results

### Results from the survey (phase 1)

Regardless of one partner’s HIV status, we found that a high level of education in either partner was associated with a lower frequency of using sexual precautions (e.g., sexual protection) during interactions that carried a risk of HIV transmission. Women from higher social class families used sexual prevention less often compared to those from modest social backgrounds. When both partners had problematic relationships with their parents, there was a decrease in the use of HIV prevention methods. Among the couples assessed, HIV-negative women exhibited behavioral traits indicative of BPD, along with histories of chronic depression and child abuse or neglect (**Table 4**).

**Table 4:** Descriptive characteristics of health behaviors in 175 HIV-discordant couples.

Characteristic	Group comparison	Sexual protection use (%)	95% CI	P-value
Male partner education	Elementary/Middle vs. High School/University	86% vs. 58%	[79.9–92.1] vs. [49.9–66.1]	*p* < 0.001
Female partner education	Same/Higher vs. Lower than male partner	60% vs. 90%	[51.4–68.6] vs. [83.9–96.1]	*p* < 0.001
Samos Syndrome background	Disadvantaged vs. Middle-high social class	87% vs. 59%	[80.6–93.4] vs. [50.1–67.9]	*p* < 0.001
Relationship with parents	Poor/problematic vs. Good	40% vs. 83%	[30.6–49.4] vs. [75.4–90.6]	*p* < 0.001

Data regarding the frequency (from ‘0% to 100%’) of sexual protection use during sexual encounters at risk of HIV transmission. Sexual protection frequency by sociodemographic characteristics. The outcome is the frequency of sexual protection during sexual encounters at risk of HIV transmission.

**Figure 1:**
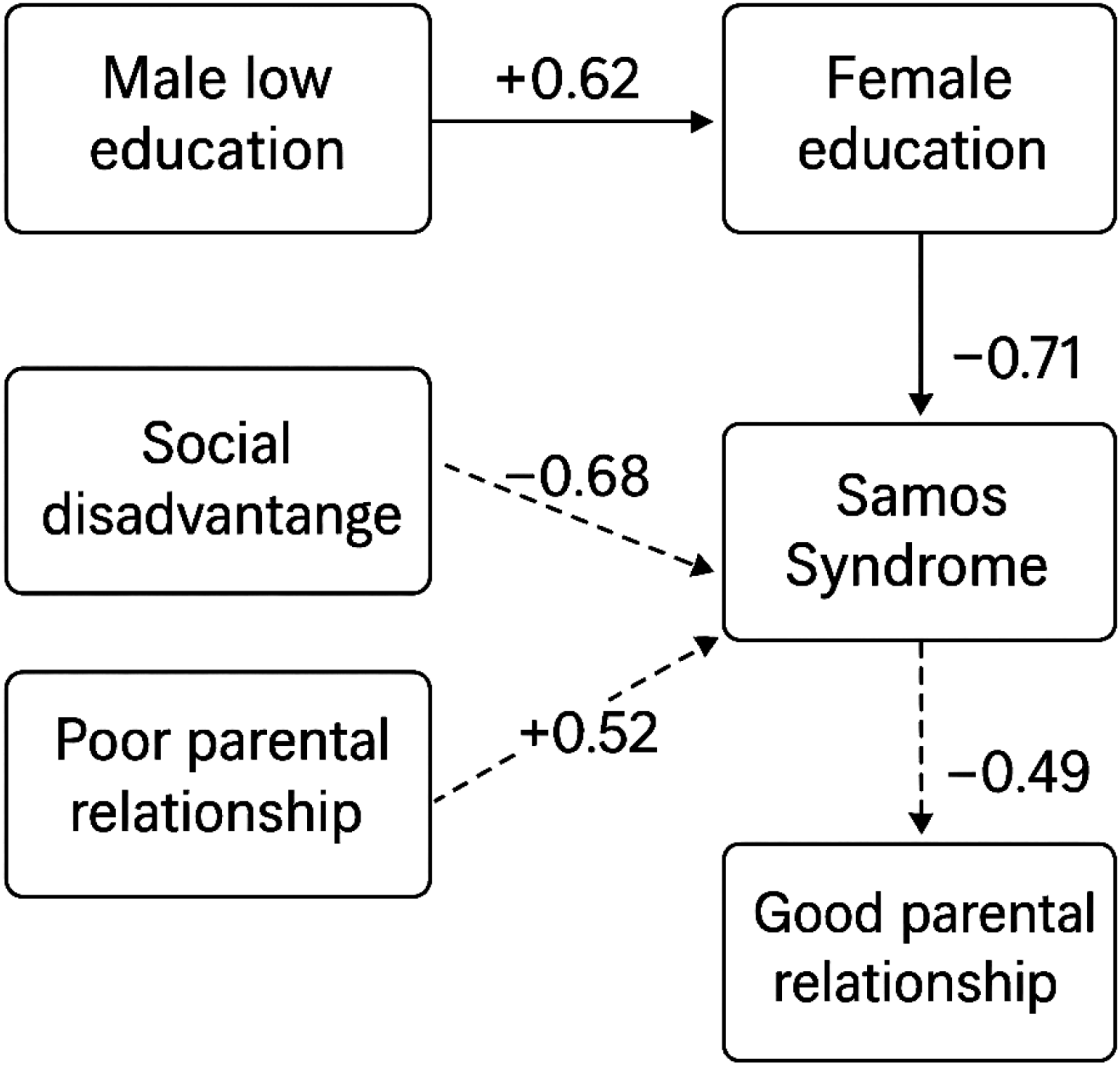
Path analysis of the quantitative study.

The analysis illustrates how educational and relational factors might shape sexual protection behaviors among HIV-serodiscordant couples. Men with lower education and women whose education was lower than their HIV-positive partners showed higher adherence to prevention. Women with Samos Syndrome from disadvantaged backgrounds also demonstrated greater sexual protection than those from higher social classes. Additionally, couples reporting at least one positive parental relationship were more consistent in using protection. These findings illustrate how structural disparities and familial dynamics might influence sexual risk-reduction behaviors in serodiscordant contexts (**Table 5**, **Figure 1**).

**Table 5:** Reinterpreted pathways linking sociodemographic factors to Samos Syndrome and sexual protection *behavior* in HIV-serodiscordant couples.

Pathway	Empirical support	Behavioral implication	Beta coefficient (β)
Male low education → Female higher education	Sexual protection use is higher (86%) when the male is less educated	May foster protective behavior but trigger Samos’ tension due to status incongruence	+0.62
Female higher education → Samos Syndrome	Sexual protection use is lower (60%) when a female is more educated	Reflects agency but may evoke relational dissonance and internalized inferiority	+0.71
Social disadvantage → Samos Syndrome	87% of female partners with Samos come from disadvantaged families	Core antecedent of the syndrome; fosters vulnerability and relational hypovigilance	+0.68
Poor parental relationship in both partners → Samos Syndrome	Sexual protection use drops to 40% with poor parental bonds	Weak relational templates during childhood in both partners impair trust (increased compliance with unhealthy behaviors) and health behavior adherence (reduced health behaviors of prevention during infectious diseases)	+0.52
Good parental relationship → Samos Syndrome	Sexual protection use rises to 83% with secure parental bonds	A positive parental bond during childhood predicts increased health behaviors in adulthood. Good parental bonding is a protective factor and a buffer against syndrome emergence	−0.49

### Qualitative narrative analysis (phase 2)

Prototypical narratives from women with BPD and from HSDHC are presented here, gathered through unobtrusive ethnographic methods and informal interviews using both structured and unstructured open-ended questions.

#### First theme: potential attraction to partners with complex health or behavioral profiles (ICD-10: BPD instability in interpersonal relationships)

This theme involved conflicting and dependent relationships between HIV-negative women with BPD and partners with complex health or behavioral profiles, aiming to address their low self-esteem. The explored path included becoming more affectionate with their stable partners and considering whether this pattern could lead to future issues such as problematic relationships, HIV or COVID-19 infection, abuse and violence from aggressive partners, and the development of potential post-traumatic stress disorder (PTSD). More women became HIV-infected, and some experienced feelings of remorse, self-blame, and suicidal thoughts in the aftermath. The typical narrative is: “I tend to fall in love and feel particularly drawn to complex partners, believing that difficulties can be overcome.”

##### Subtheme: low self-esteem and the pursuit of love (ICD-10: instability in self-image)

The typical narratives are, “During childhood, I suffered from abuse and violence. Now I have unprotected sexual relationships with my partner with HIV to prove that love is possible,” and “I do not protect myself from the virus of my partner for fear he rejects me.”

##### Subtheme: a relationship regardless (ICD-10: low self-esteem)

Common among some women with a history of trauma, violence, and BPD is the tendency to blame themselves when relationships with partners appear to fail. Some of these women often invest emotionally in male partners, and when those partners do not reciprocate, they feel guilty and abandoned. In response, they may accommodate their partners’ requests to protect the romantic relationship. However, some of these requests from partners might sometimes be problematic or, in cases involving infectious diseases, might even include asking the HIV-negative women not to protect themselves from contagious illnesses, such as requesting they refrain from using sexual protections. The typical narrative is: “Although my romantic relationship has problems and my partner is HIV positive and has issues with drugs, I do not dare to abandon him. I also feel responsible if the relationship does not work.” “Several times, he asked me not to use protection during our sexual relationships.”

##### Path analysis

In the second part of this study, we conducted a path analysis to build a theoretical model from our findings. Each narrative was then coded using a structured matrix using a 3-point ordinal scale (0 = absent, 1 = moderate presence, 2 = strong presence) and a 2-point binary scale (0 = absent; 1 = present) (**Table 6**).

**Table 6:** Thematic constructs and variable definitions.

Construct label	Description	ICD-10 Reference	Variable type	Endogenous/dependent (EN) or exogenous/independent (EX)
Partner Complexity Attraction (PCA)	Attraction to partners with health/behavioral difficulties	F60.3 (BPD – instability in relationships)	Binary / Ordinal (0=no attraction; 1 = attraction) / partner (0 = low level of difficulties; 1 = medium level; 2 = high level of difficulties).	Ex
Low Self-Esteem (LSE)	Self-deprecating beliefs, emotional dependence	F60.3 / F60.31	Ordinal (0 = very low self-esteem; 1 = medium level of low self-esteem; 2 = high level of low self-esteem)	Ex
Unprotected Intimacy (UPI)	Engagement in unprotected sex despite known risk	Z72.51 / F60.3	Binary (0 = protected; 1 = unprotected)	En
Trauma History (TH)	Childhood abuse, violence, neglect	Z61.4 / F43.1	Binary (0 = no trauma; 1 = trauma)	Ex
Self-Blame and Guilt (SBG)	Internalized responsibility for relationship failure	F60.3 / F33.2	Ordinal (0 = low, 1 = moderate, 2 = high internalized responsibility)	En
Suicidal Ideation (SI)	Thoughts of self-harm or suicide	R45.81 / F32.3	Binary (0 = no thoughts; 1 = thoughts are present)	En
Accommodation of Risk (AR)	Compliance with harmful partner requests	F60.3 / Z72.51	Ordinal (0 = nil/low, 1 = moderate, 2 = high)	En
HIV Infection Outcome (HIV+)	Seroconversion following relationship dynamics	B20 / Z21	Binary (0 = no seroconversion; 1 = seroconversion)	En

**Figure 2:**
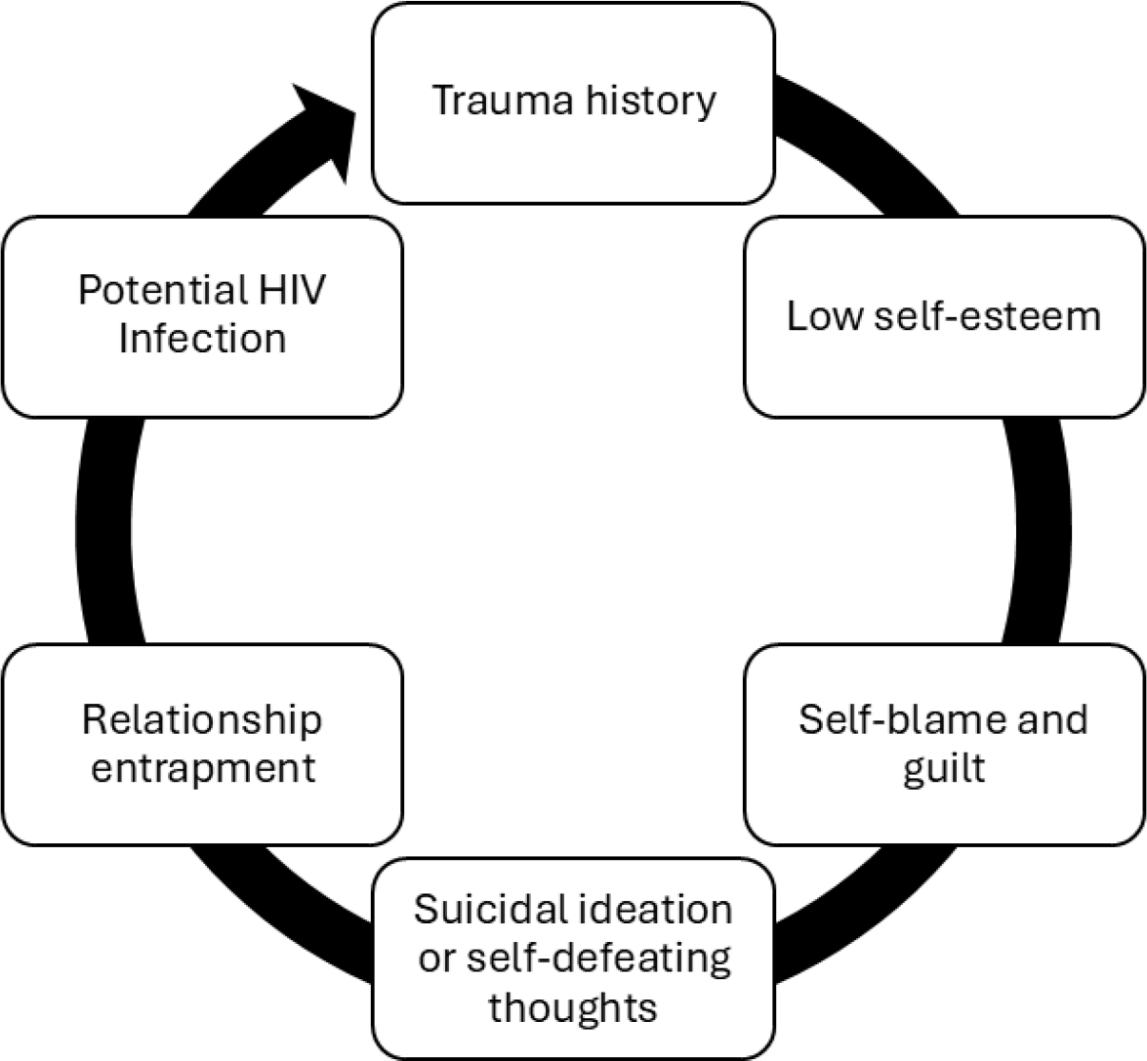
The sequence path resulting in HIV infection.

From these variables we hypothesized the path as the following: (1) Trauma History (TH) → Low Self-Esteem (LSE) → Partner Complexity Attraction (PCA) → Unprotected Intimacy (UPI) → HIV Infection Outcome (HIV+); (2) Low Self-Esteem (LSE) → Self-Blame and Guilt (SBG) → Suicidal Ideation (SI); and (3) Partner Complexity Attraction (PCA) → Accommodation of Risk (AR) → HIV Infection Outcome (HIV+). This structure enables the estimation of beta coefficients for each path, allowing for the testing of directional influence while preserving the narrative logic (**Figure 2**).

**Figure 3:**
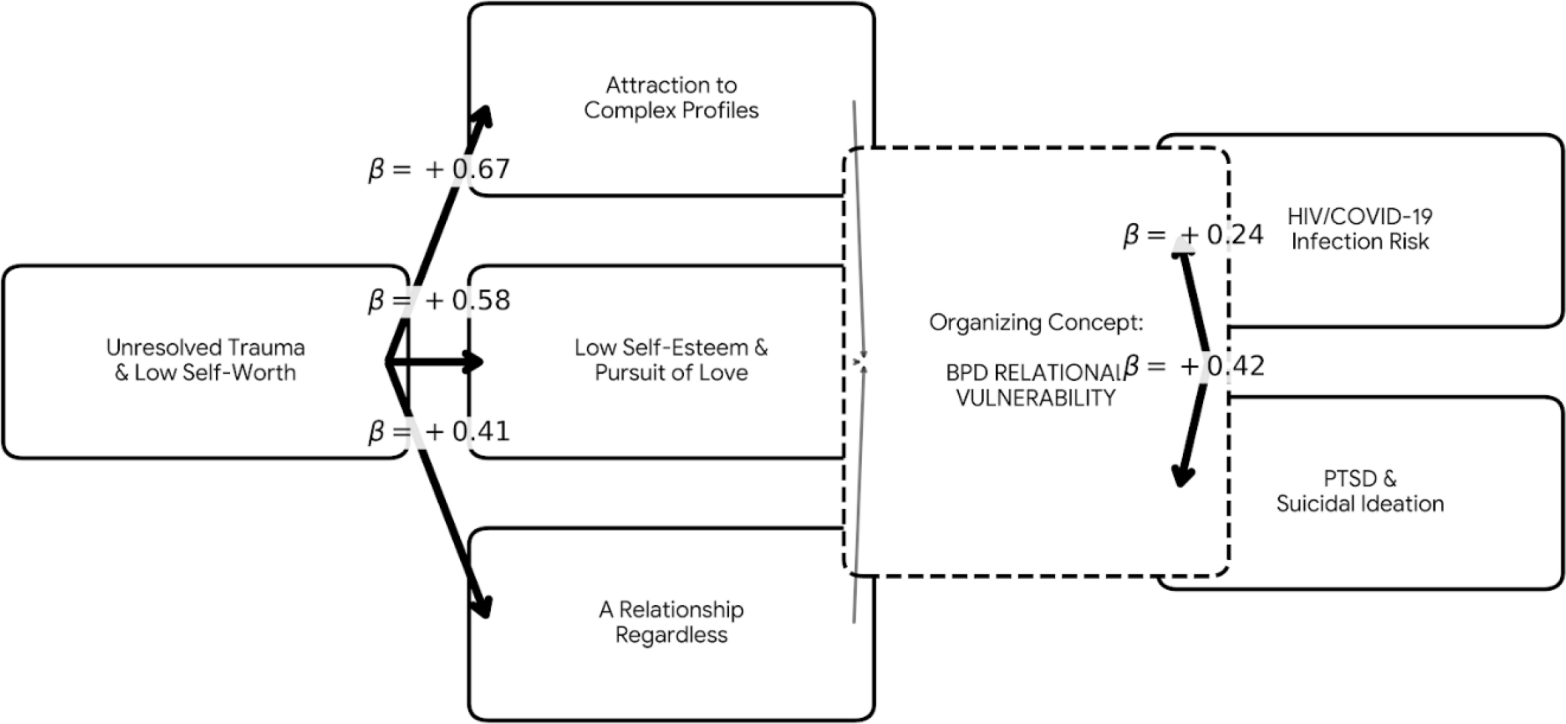
Path Analysis of BPD Relational Patterns and Health Outcomes. This structural model illustrates how unresolved trauma contributes to three thematic clusters aligned with ICD-10 criteria. The primary driver, attraction to complex relational profiles, reflects emotional dependence and idealization. When coupled with low self-esteem and persistent relational engagement, these patterns facilitate a pursuit of intimacy in which (sexual and biological) protective health behaviors may be compromised in favor of symbolic connection. These vulnerabilities are associated with increased risk for sexually transmitted infections and significant psychological consequences, including post-traumatic stress and suicidality. The model highlights the interplay between relational vulnerability and adverse health outcomes in women with BPD.

The qualitative phase revealed emotionally intense narratives among HIV-negative women with Borderline Personality Disorder (BPD) in serodiscordant relationships, organized into three thematic clusters aligned with ICD-10 criteria. The central theme, *attraction to partners with complex health or behavioral profiles*, reflected emotional dependency and idealization, often rooted in unresolved trauma and low self-worth (β = +0.67). The subtheme *low self-esteem and the pursuit of love* involved trauma reenactment, with unprotected sex used to express affection and avoid rejection (β = +0.58). The third cluster, *a relationship regardless*, captured persistence in harmful partnerships driven by fear of abandonment and self-blame (β = +0.41). These relational patterns were linked to increased risk of HIV/COVID-19 infection (β = +0.24) and severe psychological consequences, including PTSD and suicidal ideation (β = +0.42), illustrating the deep interplay between symbolic intimacy, relational vulnerability, and mental health outcomes (**Figure 3**).

#### Second theme: risk-taking behaviors (ICD-10: sex at risk)

A characteristic of this subtheme is that women with BPD tend to engage in various risk-taking (sexual) behaviors, showing a clear understanding of the health risks (such as HIV, COVID-19, and sexually transmitted diseases), social consequences (like unwanted pregnancies and exploitation by problematic partners), and psychological effects (including regret and guilt after risky behaviors). The typical narrative often becomes, “I have a partner with AIDS, but we usually do not use protection because we love each other.”

##### Subtheme: an impulsive attitude toward risk and limited ability to predict or prevent risks (ICD-10: unpredictable actions)

In this subtheme, we identified several risky behaviors that could influence the spread of infectious diseases and affect the health of individuals with BPD. Among those with a history of trauma and low mood, we observed a combination of impulsive actions and decreased awareness of their consequences. The typical stories we collected included, “During childhood, I experienced abuse and violence. Now I have unprotected sexual relationships with my partner with HIV to show that love is possible.”

##### Path analysis

In the second part of the study, we conducted a path analysis to build a theoretical model from our findings. Each narrative was then coded using a structured matrix using a 3-point ordinal scale (0 = absent, 1 = moderate presence, 2 = strong presence) and a 2-point binary scale (0 = absent; 1 = present) (**Table 7**).

**Table 7:** Thematic constructs and variable definitions.

Construct label	Description	ICD-10 reference	Variable type	Endogenous/dependent (En) or exogenous/independent (Ex)
Risk-Taking Sexual Behavior (RTSB)	Engaging in sexual activity despite known health risks	Z72.51 / F60.3	Ordinal (0−2)	En
Awareness of Risk (ARW)	Cognitive recognition of health/social consequences	Z72.6 / F60.3	Ordinal (0−2)	En
Impulsivity (IMP)	Acting without foresight or planning	F60.3 / F91.0	Ordinal (0−2)	E
Trauma History (TH)	Childhood abuse, neglect, or violence	Z61.4 / F43.1	Binary (0=no trauma; 1 = trauma)	Ex
Low Mood (LM)	Depressive symptoms or affective instability	F32.0 / F60.3	Ordinal (0−2)	Ex
Unprotected Intimacy (UPI)	Sexual contact without protection despite HIV risk	Z72.51 / B20	Binary (0 = protected, 1 = unprotected)	En
Guilt and Regret (GR)	Psychological consequences post-risk	F60.3 / F33.2	Ordinal (0–2)	En

**Figure 4:**
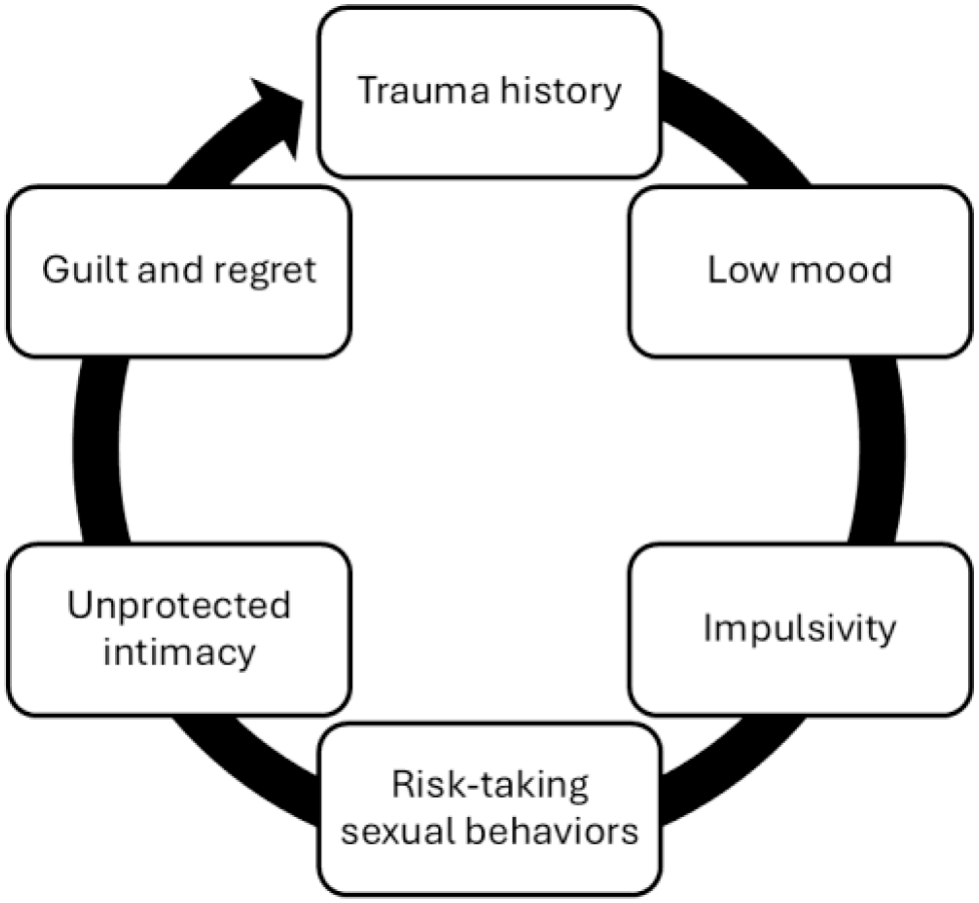
A cycle of trauma, impulsiveness, and reduced health behaviors during sexual intimacy.

Path relationships hypothesized were the following: (1) TH → LM → IMP: Trauma influences mood, which increases impulsivity; (2) IMP → RTSB: Impulsivity drives risk-taking sexual behavior; (3) RTSB → UPI → GR: Risk behavior leads to unprotected intimacy, resulting in guilt/regret (4) LM → ARW → GR: Low mood may reduce awareness of risk, which affects psychological outcomes (5) ARW moderates RTSB → GR: Higher awareness may buffer or intensify guilt (**Figure 4**).

**Figure 5:**
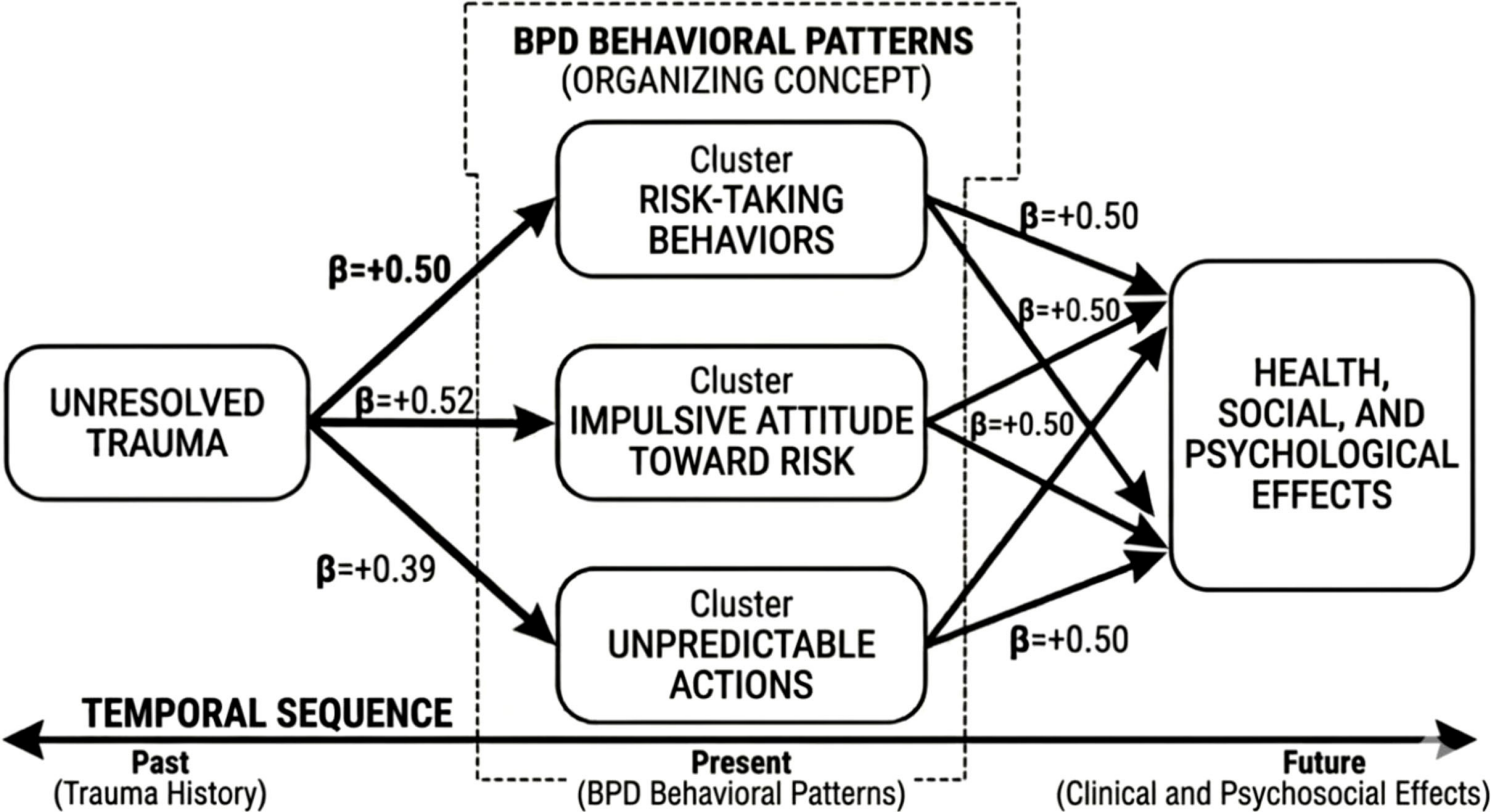
Path Analysis of the biopsychosocial cascade linked to unresolved trauma. This model illustrates how unresolved trauma drives core BPD patterns: risk-taking behaviors, impulsive attitudes toward risk, and unpredictable actions. This temporal cascade demonstrates that patients frequently prioritize symbolic acts of intimacy and emotional loyalty over physical safety, ultimately resulting in severe health, social, and psychological consequences.

Narratives from HIV-negative women with BPD in serodiscordant relationships illustrate a central theme of *Risk-Taking Behaviors* (β = +0.50), where sexual risk is consciously accepted as an expression of emotional loyalty. This paradoxical fusion of love and self-endangerment is reinforced by the subtheme *Impulsive Attitude Toward Risk* (β = +0.52), linking trauma histories and emotional dysregulation to symbolic acts of intimacy. *Unpredictable Actions* (β = +0.39) further reflect erratic behaviors that heighten vulnerability. These patterns culminate in *Health, Social, and Psychological Effects* (β = +0.50), forming a biopsychosocial cascade of illness, relational instability, and emotional distress rooted in unresolved trauma (**Table 8**, **Figure 5**).

**Table 8:** Summary of beta coefficients.

Pathway	β coefficient	Interpretation
IMP → RTSB	0.52	Impulsivity drives risk-taking sexual behavior as a symbolic act of intimacy.
RTSB → UPI	0.50	Risk-taking leads to the conscious acceptance of unprotected intimacy.
UPI → GR	0.50	Unprotected behavior culminates in psychological health effects (guilt/regret).
TH → LM	0.39	Trauma history correlates with erratic emotional states and unpredictable actions.
RI → HRE	0.81	Relationship instability is the primary driver of exposure to ultimate health risks.

Regarding the temporal sequence, this model illustrates how unresolved trauma serves as the foundational catalyst for a temporal cascade that transforms early life vulnerability into acute health risks. The process begins with trauma history influencing low mood and impulsive attitudes toward risk, which drives a shift where sexual risk is consciously accepted as a symbolic expression of emotional loyalty. As the cycle progresses, unpredictable actions and the prioritization of intimacy over physical safety lead directly to unprotected sexual behavior. This biopsychosocial sequence culminates in health risk exposure and severe psychological distress, with relational instability emerging as the most potent predictor of the outcome at a summarized coefficient of 0.81. This value represents the terminal point of caregiving and romantic pathways, indicating that once relationship instability and role confusion take hold, the statistical likelihood of high-risk exposure to infections such as HIV and COVID-19 reaches its peak.

#### Third theme: role confusion (ICD-11: inability to maintain close and mutually satisfying relationships)

##### Subtheme: replacing empathetic interactions with overinvolved relationships (ICD-11: issues in functioning related to aspects of the self and self-direction)

We observed this pattern among some professionals with BPD in healthcare who became enmeshed in their relationships with HIV+ clients. To explore the depth of the patients’ suffering, counter-transference often shifted into romantic love, risking the professional relationship due to emotional involvement. The common narrative was, “I fell in love with a person in my care because of my profession (e.g., a client or patient) who had some physical or psychological issue and was COVID-19/HIV positive.”

##### Subtheme: replacing empathetic interactions with overly involved relationships (ICD-11: Lack of accuracy about one’s strengths and limitations)

In women with BPD, there appears to be a tendency to have limited foresight about the long-term effects of romantic love. To build a satisfying relationship from the beginning, impulsiveness in choosing partners often leads to falling into violent relationships. However, these traits are evident from their very first encounters. We observed that they tend to underestimate the risks posed by others, making some women with BPD highly vulnerable to exploitation and violence from their partners. The typical narration goes, “I tend to fall in love with persons I see as needy or have AIDS, even if I get stuck in challenging stories or the classic ‘lost cause.’ These relationships make me feel helpful,” “Although my romantic relationship has problems and my partner is HIV positive and has issues with drugs, I do not dare to leave him. I also feel responsible if the relationship does not work,” “The more a person has a particular problem (e.g., physical, social, mental), the more I feel fatally attracted to him as I feel I could help improve him.”

##### Path analysis

In the second part of Phase 2 we conducted a path analysis to build a theoretical model from our findings. Each narrative was then coded using a structured matrix using a 3-point ordinal scale (0 = absent, 1 = moderate presence, 2 = strong presence) and a 2-point binary scale (0 = absent; 1 = present) (**Table 9**).

**Table 9:** Thematic constructs and variable definitions.

Construct label	Description	ICD-10 reference	Variable type	Endogenous/independent (En) or exogenous/dependent (Ex)
Boundary Erosion (BER)	Difficulty maintaining professional/personal boundaries	F60.3 / Z73.2	Ordinal (0–2) (0 = low, 1 =moderate, and 2 high difficulty)	Ex
Romantic Counter-Transference (RCT)	Emotional overinvolvement with clients due to perceived suffering	F60.3 / Z60.4	Ordinal (0–2) (0 = low, 1 = moderate, and 2 = strong emotional overinvolvement)	Ex
Rescue Fantasy (RF)	Compulsion to help or “save” vulnerable partners	F60.3 / Z73.1	Ordinal (0–2) (0 = low, 1 = moderate, 2 = strong compulsion)	Ex
Impaired Risk Appraisal (IRA)	Underestimation of relational danger or exploitation	F60.3 / Z72.6	Ordinal (0–2) ( 0 = low, 1 = moderate, 3 = strong underestimation)	Ex
Trauma History (TH)	Childhood abuse, neglect, or relational trauma	Z61.4 / F43.1	Binary (0=absent; 1=present childhood abuse)	En
Low Mood (LM)	Depressive symptoms or affective instability	F32.0 / F60.3	Ordinal (0–2) (0 = low, 1 =moderate, 2 = strong depressive symptoms)	En
Relationship Entrapment (RE)	Inability to exit harmful or exploitative relationships	F60.3 / Z63.5	Ordinal (0–2) (0 = low, 1 = moderate, 2 = strong inability)	Ex

**Figure 6:**
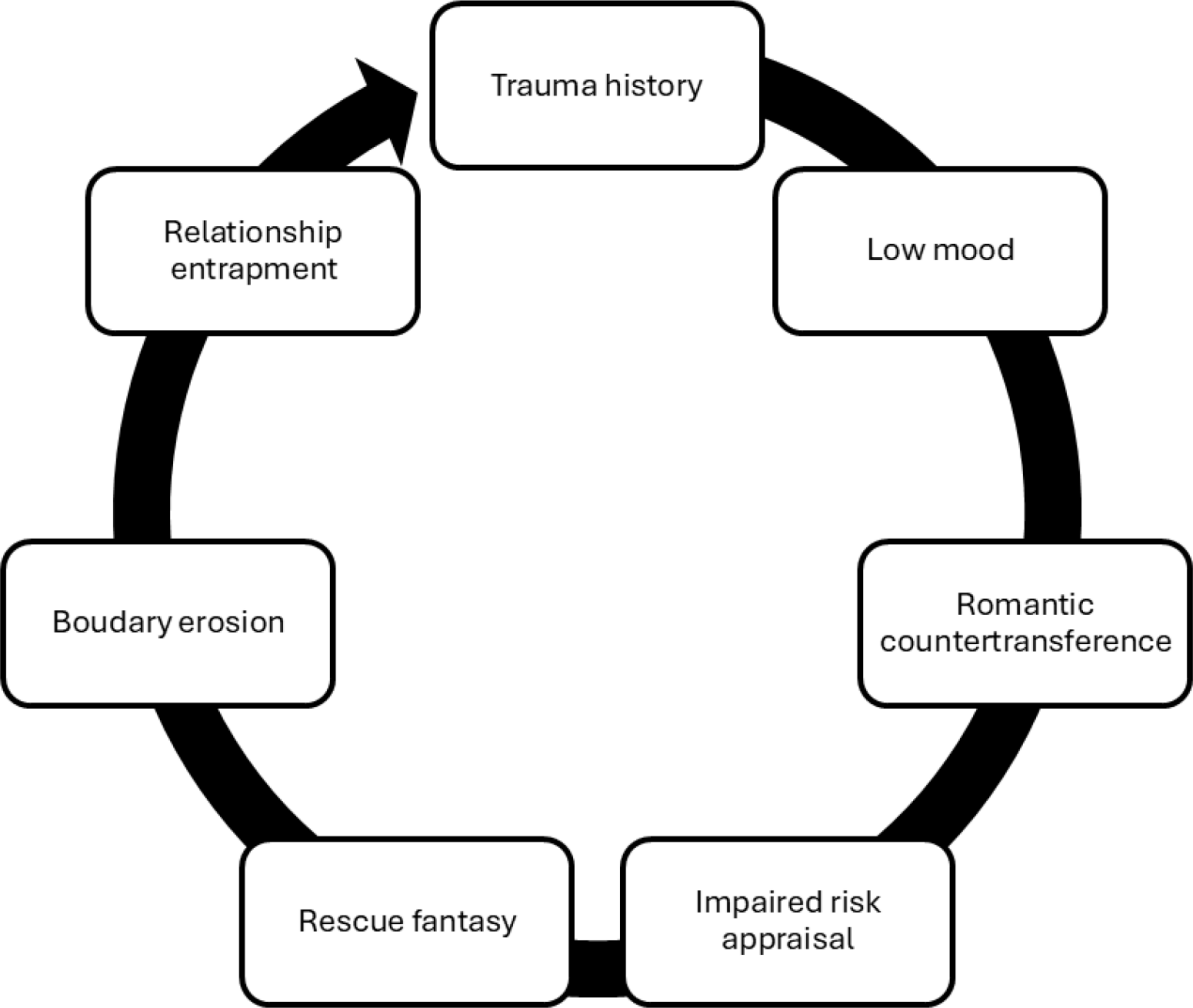
Cycle of trauma and relationship entrapment in their temporal sequence.

*The paths were hypothesized and explained as following:* (1) TH → LM → RCT: Trauma history contributes to low mood, which increases susceptibility to romantic counter-transference; (2) RCT → RF → RE: Emotional overinvolvement leads to rescue fantasies, which reinforce entrapment in dysfunctional relationships; (3) LM → IRA → RE: Depressive symptoms impair risk appraisal, increasing vulnerability to exploitation, (4) RF → BER → RE: Rescue fantasies erode boundaries, escalating relational enmeshment and entrapment; (5) IRA moderates RF → RE: Accurate risk appraisal may mitigate or exacerbate the effects of rescue-driven relational entrapment (**Figure 6**).

**Figure 7:**
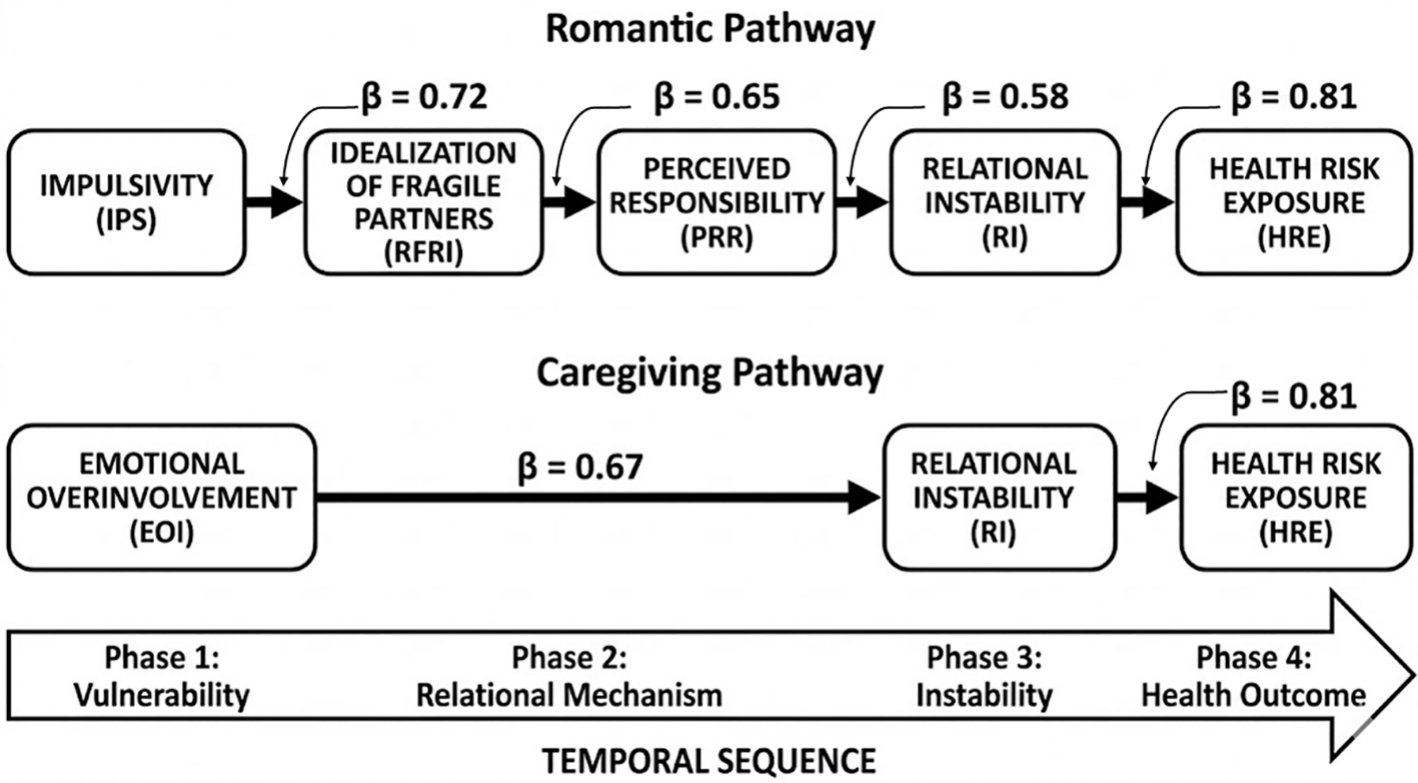
Path Analysis of the temporal sequence leading to Health Risk Exposure. This model delineates the progression from psychological vulnerability to adverse health outcomes. The Romantic Pathway begins with impulsiveness, which idealizes fragile partners and fosters a maladaptive sense of responsibility. Simultaneously, the Caregiving Pathway shows how emotional overinvolvement leads to role confusion. Both pathways converge on chronic relational instability, which serves as the primary driver of health risk exposure. The temporal sequence illustrates that the collapse of relational boundaries and stability is the critical factor increasing physiological vulnerability to infections like HIV and COVID-19.

The study developed two hypothesized pathways to illustrate how relational and psychological mechanisms contribute to health risk exposure in women with borderline personality disorder (BPD). The first pathway links impulsivity (IPS) to the idealization of fragile romantic partners (RFRI), fostering perceived responsibility (PRR), which in turn leads to relational instability (RI) and increased exposure to health risks (HRE). The second pathway focuses on emotional overinvolvement (EOI) in caregiving roles, where excessive investment results in role confusion (RI) and similarly heightens HRE. Path modelling illustrates RI and EOI as the strongest predictors of health risk (β > 0.80), supporting the ICD-11’s view of BPD as involving impaired relational functioning and highlighting the need for clinical strategies that promote relational foresight, boundary regulation, and emotional containment (**Table 10**, **Figure 7**).

**Table 10:** Illustrative Beta coefficients.

Pathway	β coefficient	Interpretation
IPS → RFRI	0.72	Strong link between impulsivity and idealization of vulnerable partners
RFRI → PRR	0.65	Idealization fosters a sense of responsibility for partner outcomes
PRR → RI	0.58	Perceived responsibility contributes to relational instability
RI → HRE	0.81	Relationship instability strongly predicts health risk exposure
EOI → RI	0.67	Emotional overinvolvement in care settings leads to role confusion
RI → HRE (from EOI pathway)	0.81	Same endpoint: unstable relationships increase vulnerability to health risks

This model illustrates how childhood trauma serves as the foundational catalyst for a temporal sequence that leads to severe health vulnerabilities in adulthood. The process begins with a history of trauma contributing to low mood and affective instability, which increases susceptibility to romantic countertransference and emotional overinvolvement. As the sequence progresses, these factors manifest as rescue fantasies—a compulsion to save vulnerable partners—while depressive symptoms impair risk appraisal and the ability to recognize relational danger. This erosion of professional and personal boundaries leads directly to relational enmeshment and a state of relationship entrapment, in which individuals are unable to exit harmful or exploitative dynamics. This biopsychosocial cascade culminates in exposure to health risks, with relational instability emerging as the most potent predictor of the final outcome, with a summarized coefficient of 0.81. This value represents the terminal point of the temporal process, indicating that once relationship instability and role confusion take hold, the statistical likelihood of high-risk exposure to infections such as HIV and COVID-19 reaches its peak.

### Theory construction (phase 3)

The overarching themes identified suggest that women who refuse preventive measures during intimate relationships at risk of HIV infection, and consequently decline any form of preventative health behavior during these risks, often have a history of child abuse and violence. We propose that individuals with BPD, as well as anyone with a background of child abuse, violence, and neglect, may develop pathophilia (from Gr: ‘attraction to illness’), a type of parasuicidal behavior, which can be understood as a form of reduced risk protection characterized by neglect of health behaviors, increased engagement in health risks, and heightened impulsive actions that can be detrimental to their health and well-being. This includes risky sexual relationships leading to STDs, sexual promiscuity, compulsive spending (such as spending beyond one’s means and compulsive hoarding), substance abuse, neglect of health behaviors related to underlying medical conditions (like high carbohydrate intake in diabetes or a sedentary lifestyle), unhealthy lifestyles (including increased social isolation and predominantly indoor living), harmful health behaviors (like anorexia, binge eating, and binge drinking), and enduring or dependent relationships with problematic partners (113, 114). According to the ICD-10 International Classification, BPD is characterized by several aspects, including unstable interpersonal relationships and life-threatening behaviors, such as engaging in risky sexual relationships (115).

#### Theory No. 1 – BPD is often comorbid with dependent personality disorder

Regarding problematic relationships, which can be psychologically or physically harmful, women with BPD may show intense dependence on others, swinging between the fear of abandonment that drives them to form all-consuming, intense relationships and the fear of being controlled by others, which causes them to end important relationships (116). The ‘relational addiction’ some women with BPD experience can become so extreme that they stay in suffer relationships, as they admit, because they want to feel helpful to their male partner, who might have social, emotional, or physical issues (117). ‘‘Interpersonal dependence’ describes the phenomenon of individuals who exhibit a notable level of reliance and a lack of individuality toward another person with whom they have romantic relationships (71). Because of this emotional reliance, they may sometimes go so far as to give up any independence and endure the heaviest sacrifices of their interests (118). We suggest that dependent personality disorder is often present alongside BPD in women, characterized by a persistent and exaggerated dependence on others for emotional and physical support, along with a fear of separation. People with DPD often act meek, clingy, and subservient; it usually begins in early adulthood and appears in various situations, often linked to unsatisfactory social interactions (119). In this context, romantic relationships are often characterized by intense interpersonal conflicts. For women with BPD who have experienced past abuse, these conflicts may unconsciously follow patterns established in previous traumatic relationships, reinforcing cycles of emotional distress and instability. Conflicts in these relationships often lead to increased suicidal ideation, especially during threats of abandonment. In the most temporary cases, parasuicidal sexual behaviors in women with BPD occur during opportunistic sexual encounters with unfamiliar individuals, sex for money to buy drugs, prostitution, or consensual encounters with people who might likely have STDs (96). Similarly, BPD health behaviors during transmissible pandemics tend to create paradoxical, unprotected social relationships at risk for contagion (72). There is a neglect of primary prevention, ignoring common sense, and a decreased concern about pandemic spread or a downgrading of awareness campaigns (96).

##### Outcomes of set theory

**Figure 8:**
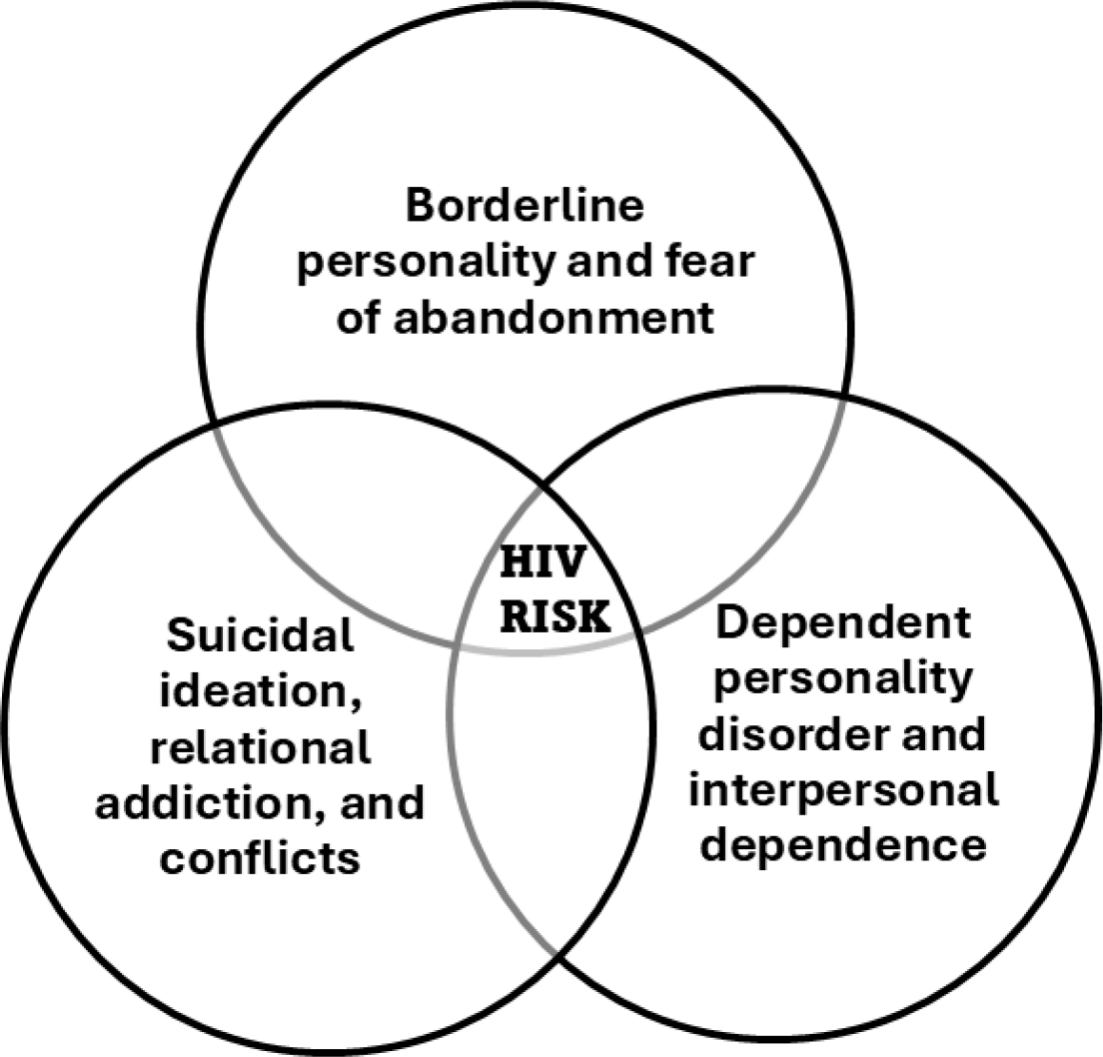
Venn diagram of confluences of the explored concepts.

In the context of Theory No. 1, we define sets *B* for Borderline Personality Disorder (BPD), *D* for Dependent Personality Disorder (DPD), *A* for Problematic Relationship Dynamics (ARD), and *S* for Suicidality and Sexual Risk Behaviors. As previously mentioned, the conceptual set of intersection between two categories or concepts is represented by the sign ‘∩‘ which identifies individuals who meet the criteria for both disorders or categories and therefore show both sets of traits simultaneously. In this case, the intersection *B*∩*D* (BPD+DPD) captures the comorbidity often observed in women with BPD, where emotional instability and fear of abandonment (from *B*) co-occur with excessive reliance and submissiveness (from *D*), forming a behavioral pattern of interpersonal dependence. When this dyad intersects with *A* (problematic relationships) as observable and present behaviors, as in *B*∩*D*∩*A*, the result is a relational configuration marked by compulsive attachment, idealization of problematic partners, and intense interpersonal conflict, traits that reinforce trauma cycles and emotional distress. This triadic overlap often leads to the emergence of *R*, a subset representing Relational Addiction, which is driven by the dialectic between the fear of being left and the fear of being controlled (*B*∩*D*∩*R* ⇒ *A*) meaning that *if B, D, A are present, then R occurs* or, if BPD, DPD and Relational Addiction occur, then problematic relationships *A* also occur. The further intersection with *S*, yielding *B*∩*D*∩*A*∩*S*, denotes the most severe clinical presentation, where suicidal ideation, parasuicidal sexual behaviors, and pandemic-related neglect of self-protection coalesce, reflecting a compounded vulnerability in women with BPD and DPD exposed to relational trauma. The whole expression becomes (*B*∩*D*∩*A∩R ⇒ S*) meaning that if B (BPD), D (DPD), A (problematic relationships), and R (relational addiction) are present, then S (suicidality or sexual *risk behaviors)* will occur. Suicidality (S) can either represent the outcome of problematic relationships or the epiphenomenon in which problematic relationships at risk for abuse and HIV infection do represent the parasuicidal behaviors. This set-theoretic model thus offers a formalized lens to interpret the complex interplay of personality pathology, abuse, and risk behavior, illustrating the need for trauma-informed, multidimensional clinical interventions (**Table 11**, **Figure 8**).

**Table 11:** Comorbidity of BDP in the context of problematic relationships.

Symbol	Set name	Description
*B*	Borderline Personality Disorder	Emotional dysregulation, fear of abandonment, impulsivity, relational instability
*D*	Dependent Personality Disorder	Excessive reliance, submissiveness, fear of separation, interpersonal dependence
*A*	Problematic Relationship Dynamics	Psychological/physical harm, relational trauma, coercive control
*R*	Relational Addiction	Compulsive attachment, self-sacrifice, and idealization of problematic partners
*S*	Suicidality & Sexual Risk Behaviors	Parasuicidal sex, opportunistic encounters, pandemic-related neglect
*T*	Trauma Reenactment Syndrome	Automatic drive to recreate elements of earlier traumatic experiences; re-victimization

#### Theory No 2. BPD is often comorbid with self-defeating personality disorder

BPD relational pathologies recall Buchli’s concept of existential aphasia, asceticism, and anorexia of life (71). In her book *Women Who Love Too Much*, Robin Norwood describes patterns of relational overinvestment, noting that some women may become so attached to an idealized version of their partner that this imagined figure becomes more emotionally significant to them than the partner as he actually is within the relationship (120). Norwood proposes that some women, including those who experience features associated with BPD, may be feeling jammed in emotionally painful or even problematic relationships because such dynamics might feel familiar or temporarily alleviate underlying distress and feelings of abandonment and loneliness (120). Norwood suggests that a vulnerability toward sadness or emotional dysregulation can lead some women with BPD to seek intensity or excitement through unstable romantic connections as a way of coping with deeper (121).

We therefore propose that BPD could be comorbid with the DSM-III-R definition of self-defeating personality disorder (SDPD), which is characterized by a persistent pattern of self-defeating behaviors that begins in early adulthood and continues across various circumstances (122). According to this diagnosis of SDPD, a person might avoid or sabotage enjoyable experiences, might be fee trapped in situations or relationships where she might suffer, and might prevent others from helping her. In the official description of SDPD this condition is indicated by at least five of the following: (1) chooses people and circumstances that lead to disappointment, failure, or mistreatment, despite better options being available; (2) disapproves of or blocks others’ efforts to offer help; (3) reacts to positive events (e.g., a new achievement) with sadness, regret, or behaviors that cause suffering (e.g., an accident); (4) provokes angry or rejecting responses from others and then feels humiliation, defeat, or pain (e.g., publicly ridicules a spouse, provoking anger, and later feels devastated); (5) declines chances for joy or hesitates to admit she is having a good time, even though she can enjoy herself and has enough social skills; (6) cannot complete tasks vital to her goals, despite having shown the ability to do so (e.g., helps classmates with writing papers but cannot write her own); (7) is indifferent to or rejects those who consistently treat her well; (8) engages in excessive self-sacrifice that is not asked for by those benefiting from it; (9) often avoids or undermines pleasurable experiences, denies herself opportunities for fun, or hesitates to admit she is enjoying herself (122). In *Daily Meditations for Women Who Love Too Much*, Robin Norwood introduces the notion of “relational dependence,” describing a pattern in which some women, here considered in relation to BPD, may remain in relationships that expose them to significant emotional or physical risk, including the possibility of contracting HIV through casual sexual encounters (121). Norwood suggests that such risks can become intertwined with the pursuit of an idealized partner whom the woman hopes to transform or support through her commitment (121).

##### Outcomes from set theory

A set-theoretically grounded narrative integrated Theory No. 2 with the linked conceptual framework visually through Venn diagrams. It preserves the symbolic logic and clinical nuance while expanding the topology to include Self-Defeating Personality Disorder (SDPD) as a fourth conceptual set. Let us define the following sets: (1) B: Borderline Personality Disorder (2) S: Self-Defeating Personality Disorder (as per DSM-III-R); (3) T: Trauma Re-enactment Syndrome (TRS); and (4) C: Complex PTSD. In this extension, we propose that the relational pathologies observed in women with BPD, particularly those involving compulsive self-sacrifice, emotional dependence, and pursuit of problematic partners, are best understood within the intersection *B*∩*S*∩*T* that is, BPD shares characteristics with suicidality, sexual risk behaviors and trauma-re-enactment disorder. This region reflects a constellation of behaviors that are not merely symptomatic but narratively structured: the woman with BPD does not simply enter harmful relationships; she reenacts unresolved trauma through relational addiction, self-sabotage, and emotional self-discipline. Robin Norwood’s concept of “loving too much” aligns with the existential aphasia described by Buchli, in which the individual experiences a disruption in authentic relational expression and engages in patterned behaviors that reflect and externalize emotional distress (118).

**Figure 9:**
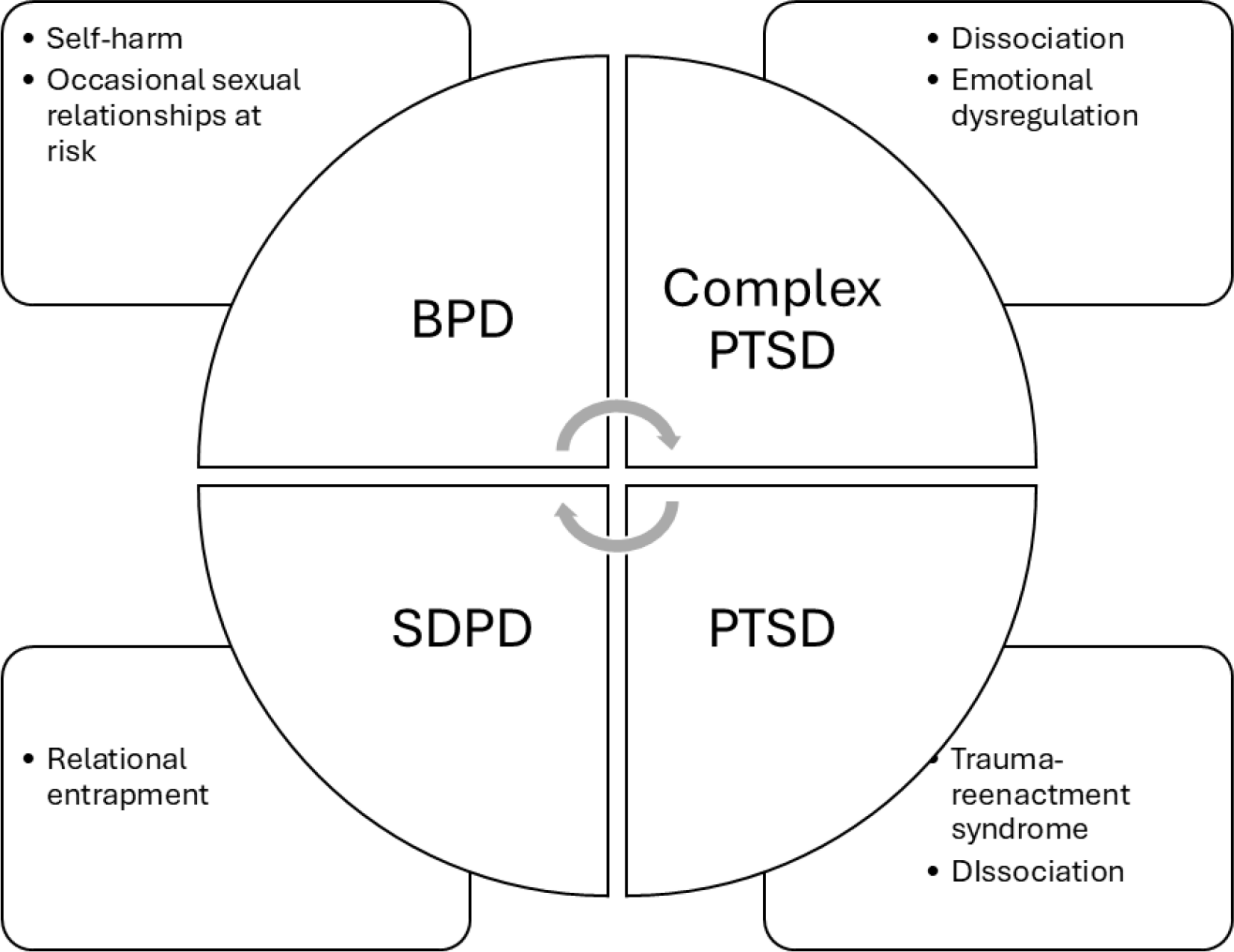
Venn diagram of confluences of the explored concepts.

In set-theoretic terms, this behavior belongs to the subset R∩B∩S∩T, where risky relationships are not avoided. Risk behaviors R (relational addiction) represent the confluence of B (borderline), S (suicidal ideation), and T (trauma reenactment). The risk of instability might potentially become a compensatory mechanism for chronic sadness, and the idealized partner, a projection of unmet childhood needs, supplants the actual partner, who remains emotionally unavailable or potentially problematic. The diagnostic criteria for SDPD (S) further reinforce this structure. Behaviors such as rejecting help, sabotaging joy, provoking rejection, and excessive self-sacrifice are all elements of subset D⊆S, where a dependent personality includes suicidality and sexual risk behaviors; this also overlaps meaningfully with B and T, borderline personality and trauma re-enactment. For example, the tendency to decline pleasurable experiences or react to success with self-punishment reflects a deep ambivalence toward self-worth, rooted in early relational trauma. This ambivalence is not confined to SDPD, but it is echoed in BPD’s emotional dysregulation and TRS’s compulsive reenactment. Thus, the union of B ∪ S ∪ T can be understood as a conceptual topology of relational dependence, in which the risk of being involved in relationships at risk reflects not a deficit in judgment but a reenactment of earlier experiences of abandonment, neglect, and emotional deprivation. Within this framework, the woman with BPD might be enduring relational difficulty as part of an internalized script that tends to equate love with sacrifice and intimacy with risk. This narrative belongs to subset A⊆B∩S∩T, meaning that problematic relationships (A) derive from the confluence of borderline personality (B), suicidality or risk behaviors (S), and trauma reenactment (T). In this case, anxious attachment and emotional dysregulation coalesce. In Norwood’s framing, the risk of contracting AIDS through casual sexual contact is not incidental; it might be part of the relational logic embedded in B∩S∩T, that is, BPD (B) shows to be comorbid or co-occurring with sexual risk behaviors (S) via trauma reenactments (T). The woman with BPD’s self-sacrifice is not merely excessive; it is structured by a belief that recovery from her condition could come through enduring suffering or pain, even, sometimes, at the cost of her health and safety. This belief reflects a dissociative distortion in reality testing, akin to what we observe in B∩C, indicating comorbidity between BPD and CPTSD, in which danger might be underestimated, and safety overestimated. By integrating SDPD into the existing framework, we expand the central intersection B∩C∩T to include S, forming a new conceptual core B∩S∩T∩C∩S, representing the comorbidity of borderline personality (B), suicidality or sexual risk behaviors (S), potentially triggered and being triggered by (double loop effect) by trauma re-enactments (T) and complex PTSD (C). This region captures the most diagnostically and phenomenologically dense zone of trauma-related psychopathology, where enduring relational conflicts, emotional dysregulation, dissociation, and self-defeating behaviors could potentially converge to create loops of reciprocal reinforcement, in this case, relationships at risk for HIV as a re-enactment of old traumas and, conversely, triggering emotional traumas in the aftermath (**Figure 9**).

#### Theory No. 3: BPD is often comorbid with complex PTSD

Dusty Miller introduced the term “Trauma Re-enactment Syndrome” in his book *Women Who Hurt Themselves* to describe women who feel compelled to injure themselves (123). Women with BPD who have experienced significant childhood trauma may, at times, become involved in relationships that are harmful or problematic. Such patterns are often understood as emerging from earlier interpersonal or familial adversity, where experiences of neglect, inconsistency, or violation shaped expectations about closeness and safety (124). Remaining in these relationships can reflect an internalized belief, formed during periods when protection or support was absent, that they are unable to safeguard themselves from harm. In adulthood, this can manifest as a repetition of familiar relational dynamics rather than a conscious choice to endure mistreatment (124).

BPD’s relationships might quickly evolve from superficial acquaintances to deep intimacy (77). Women with BPD show distinct traits in their relationships, displaying anxious attachment behaviors such as demanding compassion or help, clinging, and watching for closeness (125). According to another study, young women with BPD are more likely to have engaged in informal relationships, have had more sexual partners in the past year, and experience an earlier start to sexual activity (126). They are also more likely to experience poorer overall health, engage in risky relationships during their first sexual encounter, and participate in non-consensual sexual activity (126).

The findings from other researchers’ interpretative phenomenological analyses highlight the gap between the lived experience of self-harm to manage emotions and its classification as a dangerous behavior. Therefore, emphasizing the risks of self-harm may not effectively prevent it (127). Based on our data, we suggest that the quality of early bonds between children and their parents or caregivers influences individuals to endorse pro-life behaviors throughout their lives, while also helping them develop sufficient skills to evaluate personal risks, health risks, and risks from others (128).

Additionally, we agree with other authors that risk behavior in BPD and its assessment are emotionally dissociated through altered cognitive and metacognitive processes (129). Altered mentalization processes have been documented in the BPD literature (130). The consequence is that some women with BPD potentially experience some impairment in reality testing, especially in moments of stress, related to self-preservation against harm and dangerous events, thereby in theory risking their safety and health by underestimating dangers and overestimating safety (131). Therefore, we assert that complex and at-risk for HIV relationships in BPD might represent a form of Complex Post-Traumatic Stress Disorder (CPTSD). This condition is characterized by re-enacting past problematic relationships and a co-occurring dissociative state, which might impact the ability to accurately assess reality, especially regarding the prevention of harmful behaviors, including sexually transmitted diseases and HIV.

In the ICD-11 framework for Complex Post-Traumatic Stress Disorder, individuals may re-experience aspects of earlier traumatic events in the present, including within their interpersonal relationships. They may actively avoid internal reminders—such as distressing thoughts or emotions—as well as external cues associated with the trauma. Alongside the hypervigilance and heightened arousal also seen in PTSD, people with CPTSD often face marked difficulties regulating their emotions, experiencing either overwhelming intensity or emotional numbing. They may hold a persistently negative self-concept and encounter significant challenges in forming and sustaining healthy or satisfying interpersonal relationships (132). Although the comorbidity of CPTSD and BPD is debated and generally not accepted (133), women with BPD carry enduring effects from past traumatic relationships into new ones, often using these relationships to escape their original trauma or to transform the current life and relationships into supportive ones. Individuals with significant trauma histories may move through cycles of intense emotion, periods of dissociation, and a tolerance of harmful or distressing experiences. In some cases, these patterns can include the reenactment of earlier trauma within adult relationships, whether stable or casual. Such dynamics may increase vulnerability to unsafe situations, including a heightened risk of sexually transmitted infections when encounters occur without adequate protection or within relationships marked by instability or coercion.

##### Outcomes from set theory

**Figure 10:**
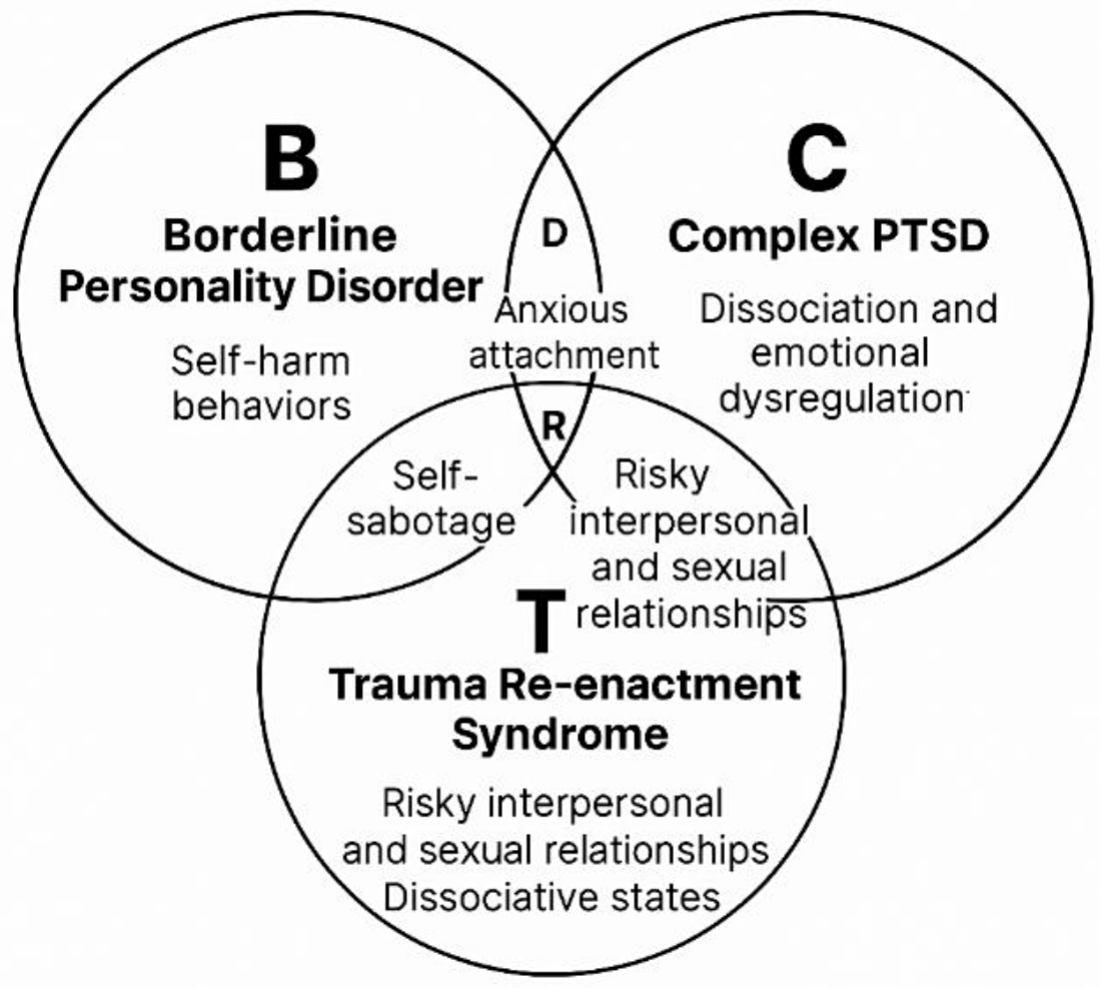
Aggregation of models and diagnoses.

A conceptual model was created to aggregate the theory into a consequential paths. The model consists of three intersecting sets: (1) *B*: Borderline Personality Disorder; (2) *C*: Complex PTSD; and (3) *T*: Trauma Re-enactment Syndrome. Each region is annotated with key clinical and behavioral phenomena: (1) *B*∩*T*: Self-harm behaviors (Subset *S*); (2) *B*∩*C*: Dissociation and emotional dysregulation (Subset *D*), anxious attachment (Subset *A*); (3) *B*∩*C*∩*T*: Risky interpersonal and sexual relationships, dissociative states, and self-defeating behaviors (Subset *R*). The intersection *B*∩*C* includes dissociation, emotional dysregulation, and unstable interpersonal relationships, features that are diagnostically distinct yet phenomenologically entangled. The overlap *B*∩*T* captures self-harming behaviors, impulsivity, and reenactment of problematic dynamics, particularly in romantic or sexual contexts. Meanwhile, *C*∩*T* encompasses negative self-concept, chronic shame, and relational avoidance or submission rooted in early trauma. The central intersection *B*∩*C*∩*T* represents a clinically rich zone of comorbidity where trauma-driven behaviors, impaired mentalization, and attachment dysregulation coalesce. Women situated within *B*∪*C*∪*T* often exhibit behaviors that are not merely symptomatic but narratively expressive reenactments of unresolved abuse encoded in relational scripts. For example, the tendency to enter unsafe sexual relationships may be interpreted as an element of *T*, but also reflects the dissociative vulnerability in *B*∩*C*, where reality testing and risk appraisal are compromised. These behaviors are not random; they are embedded in a trauma-informed logic that challenges linear diagnostic boundaries. Interpretative phenomenological analysis suggests that the clinical framing of self-harm as “dangerous” may fail to resonate with individuals whose lived experience positions such acts within *T* as attempts at emotional regulation or symbolic communication. The epistemic dissonance between clinical risk discourse and patient narrative illustrates the need for methodological pluralism when analyzing *B*∪*C*∪*T*. Moreover, early attachment disruptions, elements external to but causally linked with all three sets, shape the internal working models that govern behavior within *B* ∩*T* and *C*∩*T*. Thus, the union *B*∪*C*∪*T* does not merely represent diagnostic comorbidity, but a conceptual topology of trauma, where each subset contributes to a recursive loop of reenactment, dysregulation, and relational instability. Understanding this topology allows clinicians and researchers to move beyond categorical diagnosis toward a more nuanced, narrative-informed formulation (**Figure 10**).

### Temporal cascade of biopsychosocial risk

The events mentioned in our analysis can also be interpreted and filtered through a temporal framework as it follows.

#### Phase 1: foundations of vulnerability (the root)

The sequence begins with Unresolved Trauma History and Early Attachment Disruptions. Within the set theory framework, this is the activation of the T (Trauma Re-enactment) and C (Complex PTSD) domains. These early experiences encode “relational scripts” that might prioritize seeking proximity over seeking safety.

#### Phase 2: psychological mediators (the driver)

As these scripts interact with B (Borderline Personality Disorder) traits, they manifest as Low Mood and Emotional Dysregulation. At this stage, the psychological need for “Symbolic Intimacy” begins to override cognitive risk appraisal. This is where the BPD internal logic diverges from the clinical risk discourse.

#### Phase 3: relational mechanisms (the trigger)

A psychological distress associated with the need for proximity triggers Boundary Erosion and Relational Instability. Here, the “Romantic” and “Caregiving” pathways identified in our research become active. To prevent relational collapse, the individual may engage in “loyalty-based” behaviors that compromise personal boundaries.

#### Phase 4: terminal health risk (the outcome)

**Figure 11:**
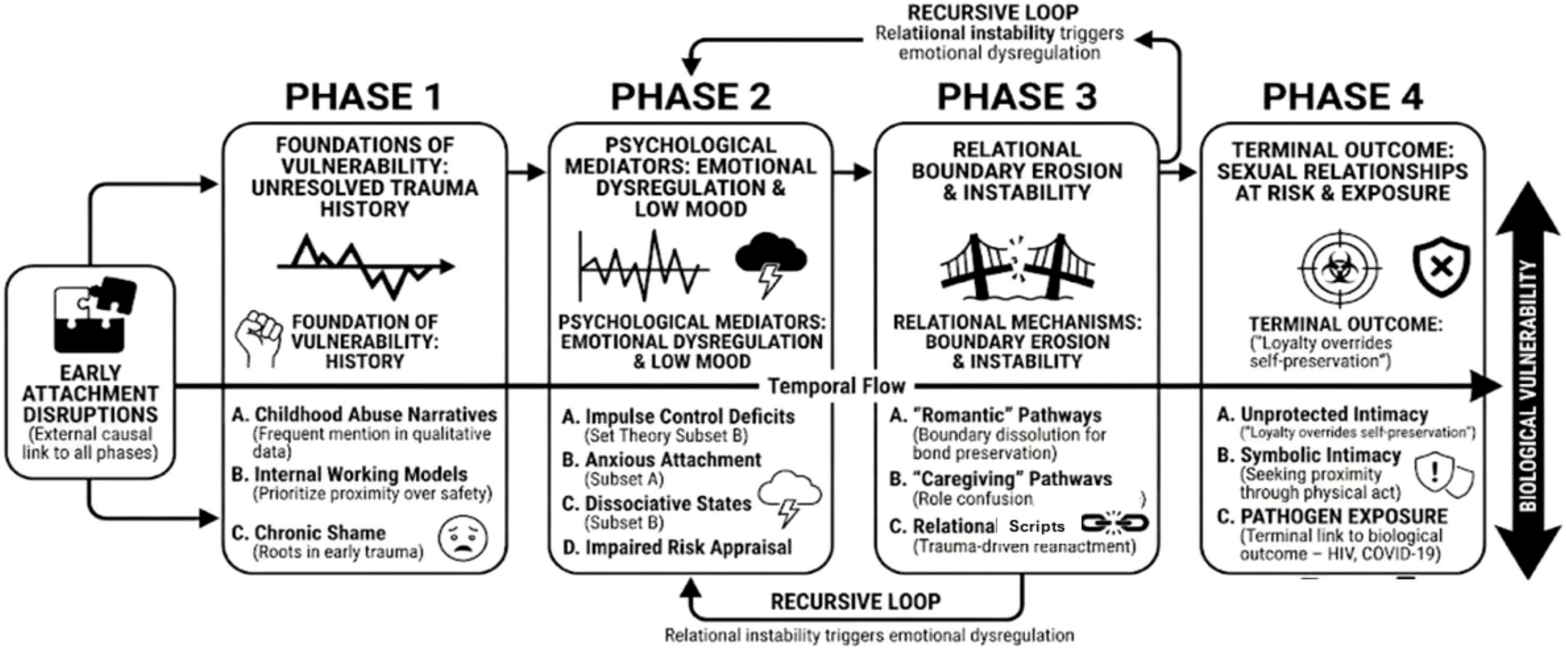
Summative image of the research findings.

The final stage of the cascade is the Sexual and Romantic Relationships at Risk phase. This is the terminal link where the psychological and social pressures culminate in actual Health Risk Exposure (e.g., exposure to HIV or COVID-19). At this point, the biological “self-preservation” instinct might be fully superseded by the trauma-informed logic of the earlier phases (**Figure 11**).

## Discussion

The synthesis of our findings reveals that the trajectory from unresolved trauma to health risk exposure is not a linear failure of logic, but a systematic biopsychosocial cascade. This research elucidates the complex mechanisms through which childhood trauma and BPD traits converge to increase physiological and psychological vulnerability in women within HIV-serodiscordant relationships. By mapping this temporal progression, we have provided an empirical framework showing how psychological distress is transformed into peculiar health behaviors within romantic relationships.

### Contribution of the research study

The primary contribution of this research is the empirical validation of a model that bridges the gap between psychiatric classifications of relational impairment and the practical realities of public health. Unlike traditional models that treat risk-taking as a purely impulsive or erratic act, this study demonstrates that for women with BPD, sexual risk (and thus risk to acquire a sexually-transmitted disease) might represent a conscious, albeit maladaptive, expression of emotional loyalty (134). By analyzing qualitative narratives through a structured lens, we provided a framework for understanding the internal logic of self-endangerment. This behavior aligns with modern views that BPD might involve some impairments in relational functioning that directly impact physical safety (135).

### New insights and novelty

Prior to this study, the link between personality disorders and infection risk was often attributed generally to a lack of self-control. However, we have learned that this connection is driven by a more nuanced progression in which relational instability serves as the critical terminal point (136). We have discovered that turmoil amid relational boundaries and the onset of role confusion are the primary drivers of health risk, driven by individual impulsivity. Furthermore, the present study highlights a paradox within symbolic intimacy, wherein some individuals with BPD may perceive unprotected sexual relationships as a necessary gesture of emotional commitment, that is, an effort to preserve a fragile bond or to fulfil a perceived role in supporting or rescuing a partner (137). This behavior indicates that the drive for emotional connection can sometimes override the biological survival instinct, rendering traditional health education, which focuses solely on cognitive awareness, somewhat incomplete.

### Impact on HIV and pandemic prevention

The findings of our study have significant implications for both HIV prevention and broader pandemic preparedness. By identifying the specific psychological “tipping points” that lead to the abandonment of protective measures, clinicians can develop more targeted interventions. Prevention strategies must move beyond teaching facts to address relational foresight, as trauma histories might impair the ability in some persons to appraise danger within intense emotional bonds (21). During global health crises, individuals with BPD could be at heightened risk not because they lack information, but because environmental stress exacerbates the very relational instability that drives risk. Recognizing the caregiving pathway allows health systems to support those who might disregard protective protocols and transmissible diseases such as HIV or COVID-19 when caring for others.

### Clinical implications and strategic interventions

The discovery of the romantic and caregiving pathways necessitates a shift toward trauma-informed infectious disease care (138). Clinical interventions must prioritize emotional containment, helping people recognize when a compulsion to omit biological protection in strict interpersonal relationships could lead risk for the own physical safety. By integrating these thematic constructs, such as boundary erosion and impaired risk appraisal, into standard intake screenings at clinics, healthcare providers can identify high-risk individuals before exposure to contagious biological agents occurs. Breaking this sequence requires a dual approach: preventing the physical risk while providing the psychological tools to manage the subsequent emotional fallout, thereby preventing a relapse into further high-risk cycles.

## Conclusions

Borderline personality disorder is frequently encountered in both inpatient and outpatient psychiatric settings, yet several clinically significant domains often remain insufficiently explored in routine assessments. In particular, the ways in which women with this diagnosis navigate sexual health, sustain intimate partnerships, and engage in behaviors that promote or compromise well-being are not always systematically evaluated. The present study emphasizes that patterns of self-defeating or health-risking behavior constitute an important clinical dimension of BPD and should be considered when assessing vulnerabilities related to sexual health decision-making and relational stability. By examining romantic relationships that involve heightened risk of HIV transmission, the study brings to light the extent to which BPD may coexist with dependent personality traits, self-defeating interpersonal patterns, and features associated with complex PTSD. These co-occurring conditions often become most visible within intimate relationships, where attachment disruptions, trauma-related reenactment, and difficulties in emotional regulation converge. For some women with BPD, these relational contexts may inadvertently increase exposure to health and emotional risks, reflecting not only symptom expression but also deeply embedded relational scripts shaped by earlier experiences of neglect, abandonment, or emotional deprivation. Understanding these dynamics highlights the need for more comprehensive and trauma-informed assessments that recognize how relational patterns, internalized narratives, and early attachment disruptions interact within the broader topology of BPD and its common comorbidities. Such an approach allows clinicians and researchers to move beyond categorical diagnosis toward a more nuanced formulation that accounts for the complex interplay between trauma, relational functioning, and health-related behaviors.

## Limitation

This study has several limitations. The first relates to the characteristics of qualitative and exploratory research findings, which are closely tied to the practice settings. Therefore, the results are primarily transferable but not generalizable. The second aspect concerns the nature of the phenomena observed and the conclusions drawn from our interpretations. Although other studies support our findings, ongoing debates about comorbidities in BPD persist, with some authors tending to categorize them according to specific models. Finally, we developed our theories by retrospectively reviewing our past findings and did not verify the accuracy of our conclusions with major stakeholders.

## Data Availability

The original contributions presented in the study are included in the article/supplementary material, further inquiries can be directed to the corresponding author/s.

## Appendix Semi-structured interview questionnaire: Samos syndrome and relational health behaviors in HIV-serodiscordant couples

1. Educational status and relational dynamics.

- What level of education have you completed?
- What level of education has your partner completed?
- How do differences in education between you and your partner affect decisions about sexual protection?
- Have you ever felt tension or imbalance in your relationship because of differences in education or status?
- Do you feel that educational differences influence how you and your partner communicate or trust each other?

2. Female education and relational agency.

- In your experience, how does a woman’s education affect her role in a relationship?
- Have you ever been in a relationship where the woman was more educated than the man? If so, how did that affect the relationship?
- When the woman is more educated, do you think it becomes easier or harder to discuss and agree on sexual protection?
- Have you ever felt uncomfortable or inferior in a relationship because of differences in education?

3. Social disadvantage and vulnerability.

- What was your family’s financial situation like when you were growing up?
- Do you think coming from a disadvantaged background affects how you approach romantic relationships?
- Have you ever felt less confident or more vulnerable in a relationship because of your social background?
- Do you think people from disadvantaged backgrounds are more likely to take risks in relationships?

4. Parental relationship quality and relational templates.

- How would you describe your relationship with your parents during your childhood?
- How would you describe your partner’s relationship with their parents?
- Do you think your relationship with your parents affects how you trust others in adulthood?
- Have you noticed that your childhood experiences influence your decisions about sexual health, like sexual protection?

5. Protective factors and resilience.

- What parts of your upbringing helped you build healthy relationships as an adult?
- Do you think having a good relationship with your parents helped you avoid unhealthy relationship patterns?
- What helps you feel safe and respected in a relationship?
- Can you think of a time when you or your partner made a choice to protect your health during a relationship?

## Glossary

AIDS: Acquired Immunodeficiency Syndrome
BPD: Borderline Personality Disorder
COVID-19: Coronavirus Disease 2019
CPTSD: Complex Post-Traumatic Stress Disorder
DPD: Dependent Personality Disorder
HIV: Human Immunodeficiency Virus
HSDHC: HIV-Serodiscordant Heterosexual Couples
NPC: Non-Prevention Couples
SDPD: Self-defeating Personality Disorder
STD: Sexually Transmitted Disease
